# Abnormal β‐Hydroxybutyrylation Modification of ARG1 Drives Reprogramming of Arginine Metabolism to Promote the Progression of Colorectal Cancer

**DOI:** 10.1002/advs.202502402

**Published:** 2025-07-11

**Authors:** Chuman Lin, Zhiyang Li, Xiaotong Zhu, Wanbing Zhou, Xiansheng Lu, Jiali Zheng, Jie Lin

**Affiliations:** ^1^ Department of Pathology Nanfang Hospital School of Basic Medical Sciences Southern Medical University Guangzhou Guangdong 510515 China; ^2^ Guangdong Province Key Laboratory of Molecular Tumor Pathology Guangzhou Guangdong 510515 China

**Keywords:** β‐hydroxybutyrylation, ARG1, arginine metabolism reprogramming, colorectal cancer

## Abstract

The abnormal arginine metabolism is characteristic of tumor cell metabolism in colorectal cancer (CRC). However, the mechanisms underlying arginine metabolic reprogramming and how altered metabolism in turn enhances CRC tumorigenicity are poorly understood. Protein post‐translational modifications (PTMs) are crucial for regulating protein function, activity, and interactions. Here, the study reports that arginine levels are elevated in CRC, accompanied by the high expression of arginase‐1 (ARG1) but low levels of ARG1 β‐hydroxybutyrylation (Kbhb) and its oncogenic role in CRC in a catalytic‐activity‐independent manner. Mechanistically, low‐level ARG1‐Kbhb‐induced arginine metabolic reprogramming by decreasing the interaction of ARG1 with SLC3A2 in CRC cells inhibits the efflux of arginine, thereby increasing intracellular arginine levels to promote tumorigenicity. P300 is identified as the “writer” of Kbhb. Inducing ARG1‐Kbhb at the Lys313 residue by β‐hydroxybutyrate (BHB) promotes the interaction of ARG1 with SLC3A2, resulting in the efflux of arginine in CRC cells. Together, these findings reveal valuable insights into arginine metabolism reprogramming involving the ARG1‐Kbhb/P300/SLC3A2 signaling axis, thereby bridging the connection between metabolic reprogramming and PTMs, which may shed light on the therapeutic potential of combining BHB with ARG1 inhibitor through the conventional enzymatic role and nonenzymatic metabolic function of ARG1 for CRC.

## Graphical abstract

Alterations in arginine metabolism and β‐hydroxybutyrylation (Kbhb) modifications play a critical role in tumor progression. This study reveals valuable insights into arginine metabolism reprogramming involving the ARG1‐Kbhb/P300/SLC3A2 signaling axis and uncovers an effective therapy combining BHB with ARG1 inhibitor through conventional enzymatic and nonenzymatic metabolic functions of ARG1 for colorectal cancer, thereby bridging the connection between metabolic reprogramming and post‐translational modifications.

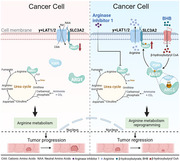

## 1 Introduction

Colorectal cancer (CRC) ranks as the second most prevalent malignant tumor and stands as the fourth leading cause of cancer‐related fatalities on a global scale.^[^ ^1^ ^]^ Although the primary treatment options include surgery, chemotherapy, and radiotherapy, the 5‐year relative survival rate of CRC patients has not improved in recent decades.^[^ ^2^, ^3^ ^]^ Therefore, gaining insights into the molecular mechanisms of CRC is crucial for the development of precise and effective treatments.

Metabolic reprogramming is one of the hallmark features of cancer cells, during which characteristics of disruptions in amino acid metabolism in ways that can meet their increased demand for amino acids, glucose metabolism, the tricarboxylic acid cycle, fatty acid metabolism, and nucleotide synthesis.^[^ ^4^, ^5^, ^6^, ^7^, ^8^ ^]^ Arginine is a highly versatile amino acid. Besides its role as a building block in protein synthesis, it is a precursor for polyamines, creatine, and nitric oxide. Arginine can also be interconverted with proline and glutamate and can promote cell growth by activating mTORC1.^[^ ^9^ ^]^ Furthermore, there is evidence that arginine impacts metabolism.^[^ ^10^, ^11^, ^12^, ^13^ ^]^ Arginine metabolism is hyperactive in CRC, with key enzymes such as arginase (ARG), endothelial nitric oxide synthase (eNOS), argininosuccinate synthetase, and ornithine decarboxylase (ODC) being overexpressed.^[^ ^14^, ^15^, ^16^ ^]^ Moreover, arginine transporters like CAT‐1 and SLC6A14 are overexpressed in CRC cells, increasing intracellular arginine levels, which supports cancer cell growth.^[^ ^16^ ^]^ Arginase 1 (ARG1) is an enzyme catalyzing the conversion of arginine to ornithine and urea. Studies have shown that ARG1 is significantly overexpressed in CRC tissues compared to noncancerous tissues. This overexpression is associated with advanced tumor stages (III‐IV), lymph node metastasis, and lower plasma albumin levels. High ARG1 expression correlates with poor overall survival (OS) and disease‐free survival (DFS), making it an independent prognostic factor for poor outcomes.^[^ ^17^, ^18^ ^]^ Given the above, we sought to investigate the role of ARG1 and arginine metabolism in CRC.

Post‐translational modifications (PTMs) are crucial biochemical processes that modify proteins after their synthesis, impacting their function, stability, and interactions.^[^ ^19^ ^]^ In addition to the direct integration of amino acids and their derivatives in metabolic reprogramming processes, amino acids also participate in mediating epigenetic regulation and post‐transcriptional modification.^[^ ^20^ ^]^ Mass spectrometry analysis in a previous study showed that ARG1 might be β‐hydroxybutyrylated, induced by β‐hydroxybutyrate (BHB).^[^ ^21^ ^]^ BHB, a substrate for the modification of lysine residues, results in the emergence of a novel form of epigenetic modification called β‐hydroxybutyrylation (Kbhb), whose modification directly regulates metabolic enzymes, regulating upstream molecules and downstream metabolic products.^[^ ^21^, ^22^, ^23^ ^]^ Besides, accumulating studies have substantiated the intricate association of Kbhb with the carcinogenesis and tumorigenesis of various human cancers, including lung adenocarcinoma (LUAD), hepatocellular carcinoma (HCC), osteosarcoma, and CRC.^[^ ^21^, ^24^, ^25^, ^26^ ^]^ However, the mechanisms underlying interactions between oncogene and tumor suppressor gene alterations, metabolic reprogramming, and epigenetic modification in cancer, as well as the full extent of their profound impact on tumor progression through abnormal crosstalk, remain unknown. Here, we focus on the role of arginine metabolism in CRC progression and explore whether ARG1 and its Kbhb modification are involved in regulating arginine metabolism and its underlying mechanisms.

## 2 Results

### 2.1 Elevated Arginine Levels and Increased ARG1 Expression in CRC

**Figure 1:**
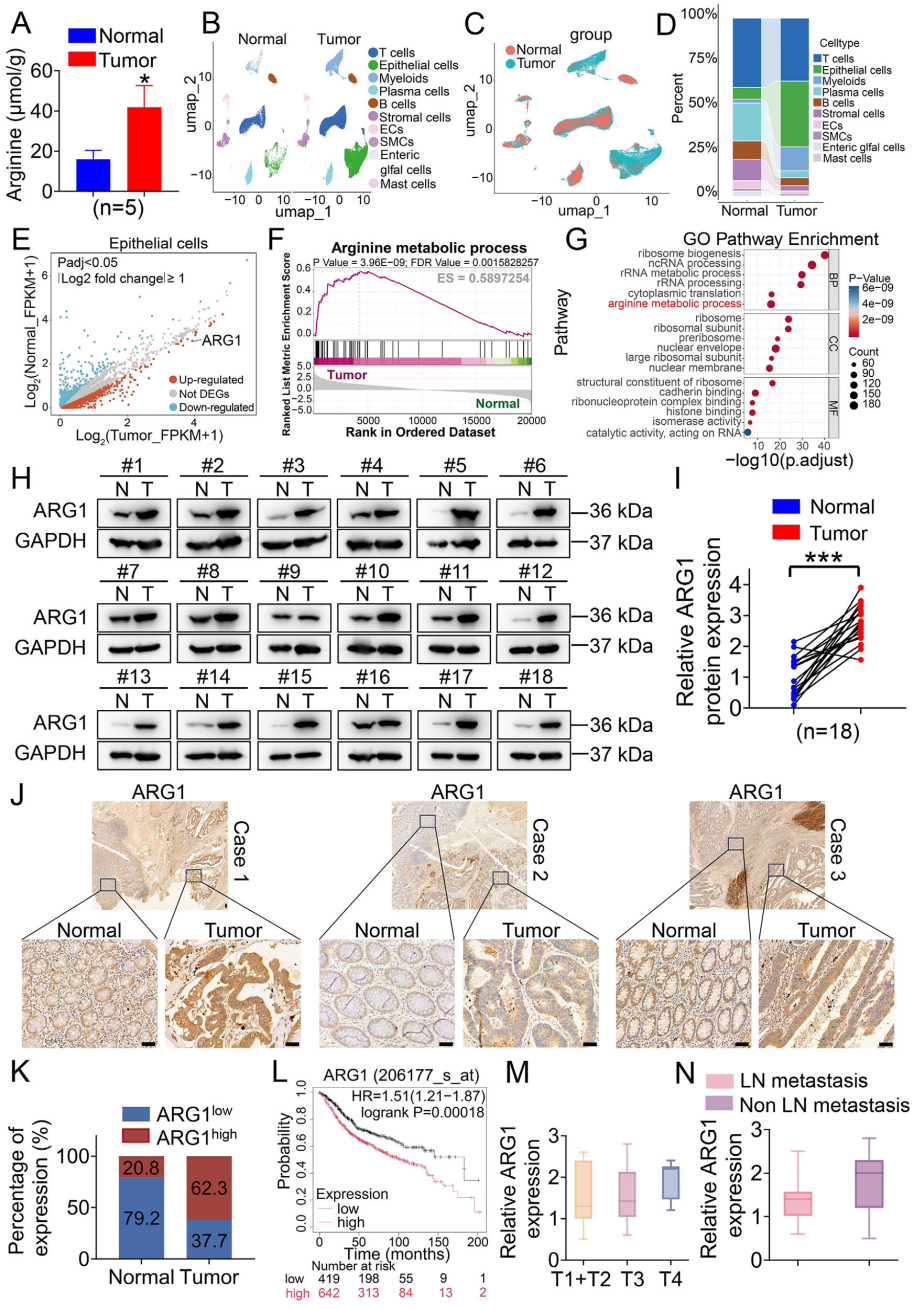
Arginine levels and ARG1 were found to be upregulated and associated with the poor overall survival of CRC. A) The content of arginine in fresh primary CRC specimens with paired noncancerous colorectal tissues respectively. n=5. B) ScRNA‐seq analysis from 23 Korean patients with primary CRC and 10 matched normal mucosa samples (GSE132465). The cluster was analyzed by using the Uniform Manifold Approximation and Projection (UMAP) method, and populations were identified by color. C) Combined clustering of normal cells and tumor oncocytes. D) Proportion distribution of cell types in normal and tumor samples. E) Scatterplots comparing DEGs between normal and tumor samples. Red dots mark genes up‐regulated in the tumor group, blue dots mark down‐regulated ones, and gray dots mark genes with no significant change between groups. The criteria for selecting DEGs: Padj<0.05 and |Log2 fold change|≥1. F) The GSEA enrichment plot showing arginine metabolic process enriched in tumor samples. G) Enriched functional terms in the GO analysis of up‐regulated DEGs in epithelial cells from tumor samples. The X‐axis (−log10(p.adjust)) represents the significance level of the enrichment analysis. H) ARG1 protein expression in normal mucosa samples (N) and primary CRC tissues (T). n=18. I) Relative protein expression of ARG1 in normal mucosa samples (N) and primary CRC tissues (T). J) Immunohistochemical detection of ARG1 expression in normal mucosa samples (N) and primary CRC tissues (T). Scale bar: 50 µm. K) Quantitative analysis of ARG1 expression in normal mucosa samples (N) and primary CRC tissues (T). n=53. L) Overall survival of ARG^high^ and ARG1^low^ patients from the Kaplan–Meier Plotter database. n=1061. Affy ID: 206177_s_at. M) T staging based on ARG1 levels. n=53. N) Lymph node metastasis based on ARG1 levels. n=53. Data are presented as means ± SD; ^*^ *P* < 0.05, ^***^ *p* < 0.001, versus the Normal group.

To confirm effects on arginine metabolism, we measured individual arginine levels in fresh primary CRC specimens with paired noncancerous colorectal tissues. As expected, arginine levels were elevated in CRC compared to paired noncancerous CRC tissues (**Figure** **1**A). To identify arginine metabolic alterations in CRC, we integrated single‐cell RNA sequencing (scRNA‐seq) data from 23 Korean patients with primary CRC and 10 matched normal mucosa samples (GSE132465) to determine whether a developmental disorder is associated with CRC. After dimensionality reduction, UMAP‐based cell clustering identified epithelioid, lymphoid, and myeloid cell clusters (Figure 1B–D). We compared transcriptional profiles to identify differentially expressed genes (DEGs) between normal mucosa samples and primary CRC tissues. We found that ARG1 expression was notably elevated in CRC tissues compared to normal mucosa tissues (Figure 1E). Gene set enrichment analysis (GSEA) and gene ontology (GO) enrichment analysis showed cell‐type‐specific and common biological functions and pathways. Arginine metabolic activity pathway enrichment was observed in cancer cells (Figure 1F,G). To further identify whether the altered gene expression corresponded to changes at the protein level, we conducted western blot and IHC staining. Consistent with the results from scRNA‐seq analysis, substantially elevated expression levels of the ARG1 protein were observed in primary CRC tissues compared with paired noncancerous colorectal tissues (Figure 1H–K). Besides, high expression of ARG1 was associated with poor overall survival (Figure 1L). However, there was no correlation between ARG1 expression and pathological characteristics, including TNM stage, lymph node metastasis, and vessel invasion (Figure 1M,N; Figure S1A, Supporting Information). Together, these results indicated that arginine metabolism was activated, and ARG1 may be an important factor in CRC.

### 2.2 ARG1 Accelerates CRC Cell Proliferation, Migration, and Invasion

**Figure 2:**
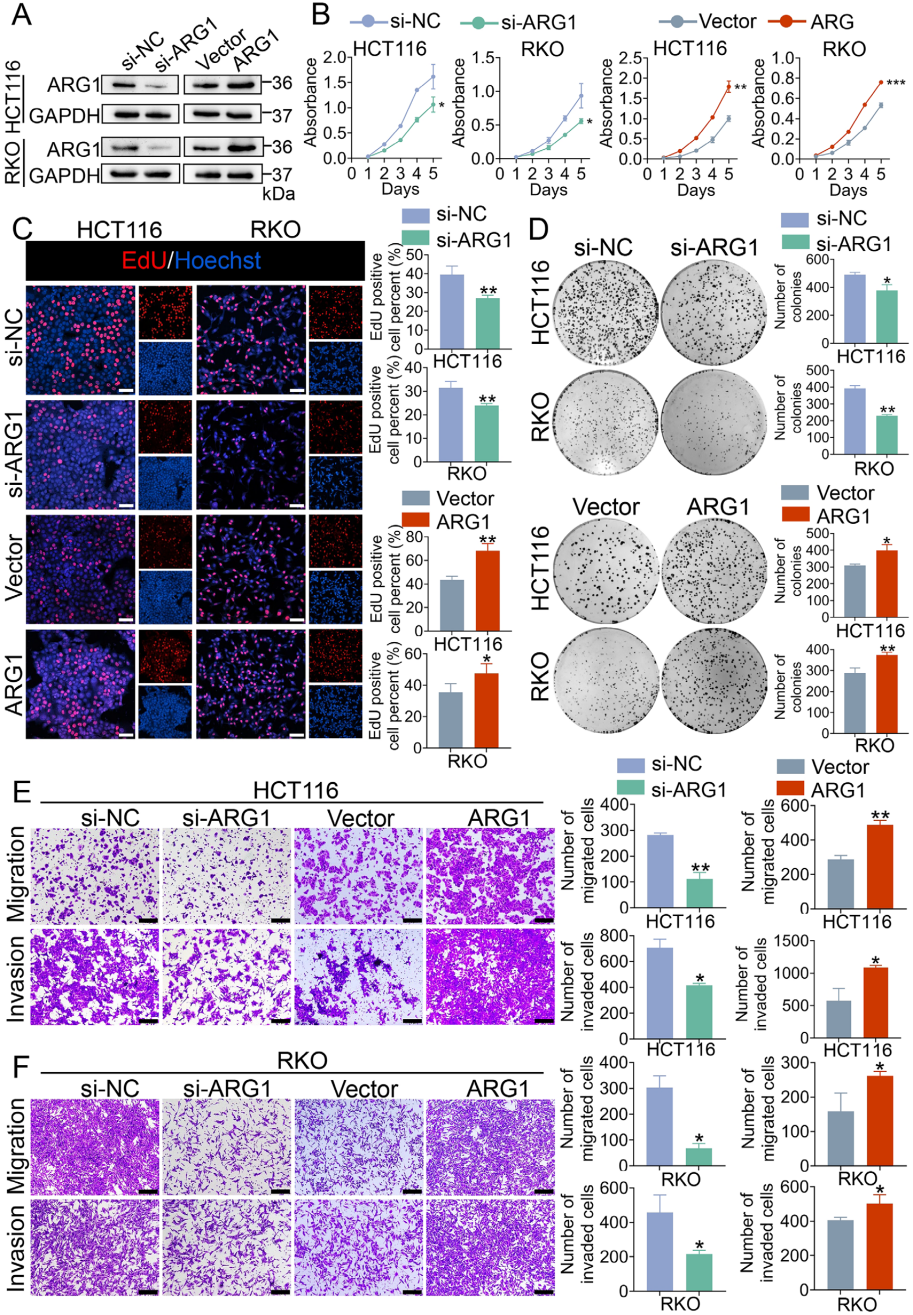
ARG1 Accelerates CRC Cell Growth and Metastasis. A) Western blot of ARG1 expression in the indicated infected cells. B) CCK‐8 assays in ARG1 knockdown or overexpressing HCT116 and RKO cells. C, D) EdU assays (C) and colony formation assays (D) in ARG1 knockdown or overexpressing HCT116 and RKO cells. The right panel showed the statistics. Scale bar: 50 µm. E, F) Transwell migration and invasion assays in ARG1 knockdown or overexpressing HCT116 (E) and RKO (F) cells. The right panel showed the statistics. Scale bar: 100 µm. Data are presented as means ± SD; n=3. ^*^ *p* < 0.05, ^**^ *p* < 0.01, ^***^ *p* < 0.001, versus the si‐NC or Vector group.

Application of western blot revealed that ARG1 expression in CRC cells was basically higher than that in normal colorectal epithelial cells NCM460 (Figure S2A, Supporting Information). To determine the biological functions of ARG1 in CRC, we applied small‐interfering RNAs (siRNAs) against ARG1 to transiently knock down ARG1 or an ARG1 overexpression vector to force expression of ARG1 in HCT116 and RKO cells (**Figure** **2**A; Figure S2B,C, Supporting Information). The results showed that ARG1 knockdown significantly decreased cell viability, proliferation ability, and colony formation ability in HCT116 and RKO cells. Inversely, ARG1‐overexpressing cells showed stronger proliferative capacity than the negative control cells (Figure 2B–D). Moreover, the migration and invasion abilities in the ARG1 knockdown group were also dampened, as indicated by transwell assays, whereas ARG1‐overexpressing cells showed higher invasive and migratory abilities than negative control cells (Figure 2E,F). Taken together, these data demonstrated that ARG1 acted as an oncogene during CRC progression.

### 2.3 ARG1 Promotes CRC Progression in a Catalytic‐Activity‐Independent Manner

**Figure 3:**
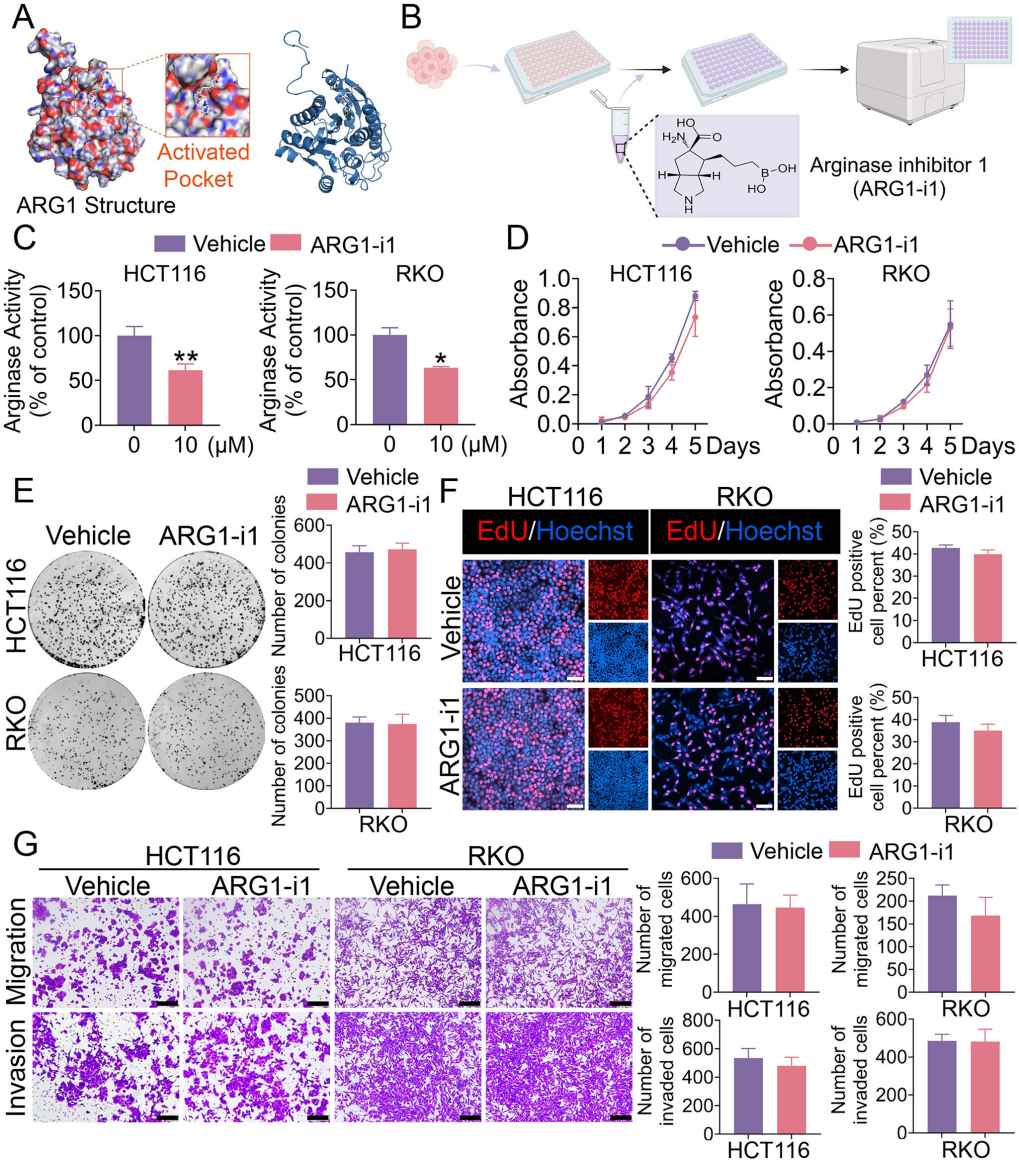
ARG1 enhances CRC progression independent of catalytic activity. A) Docking models were performed based on the activated pocket of the ARG1 crystal structure and the ARG1‐i1 compounds. B) Schematic of HCT116 and RKO cells supplemented with or without ARG1‐i1, followed by Arginase Activity Assay. Created in BioRender. Lin, C. (2025) https://BioRender.com/vw2bmq9. C) Arginase activity in HCT116 and RKO cells treated with or without ARG1‐i1. D) CCK‐8 assays were performed to assess the proliferation of HCT116 and RKO cells treated with or without ARG1‐i1. E, F) EdU assays (E) and colony formation assays (F) were performed in HCT116 and RKO cells treated with or without ARG1‐i1. The right panel showed the statistics. Scale bar: 50 µm. G) Transwell migration and invasion assays were performed in HCT116 and RKO cells treated with or without ARG1‐i1. The right panel showed the statistics. Scale bar: 100 µm. Data are presented as means ± SD; n=3. ^*^ *p* < 0.05, ^**^ *p* < 0.01, versus the Vehicle group.

To explore whether ARG1‐induced CRC progression is dependent on the enzymatic activity or nonenzymatic function of ARG1, we used arginase inhibitor 1 (ARG1‐i1) and conducted CCK‐8 assays, EdU assays, colony formation assays, transwell migration and invasion assays (**Figure** **3**A). We further validated in CRC cells using CCK‐8 assay (Figure 3B). Intriguingly, ARG1‐i1 diminished ARG1 enzymatic activity but failed to impair cellular proliferation and colony formation ability (Figure 3C–F). Consistently, we did not observe any significant alterations in migration and invasion abilities upon ARG1‐i1 treatment (Figure 3G). Together, these results suggested that ARG1 serves as an oncogenic regulator in CRC progression independently of its catalytic activity.

### 2.4 ARG1 is Lowly β‐Hydroxybutyrylated in CRC and BHB Inhibits the Growth of CRC Cells by Regulating the Kbhb of ARG1

**Figure 4:**
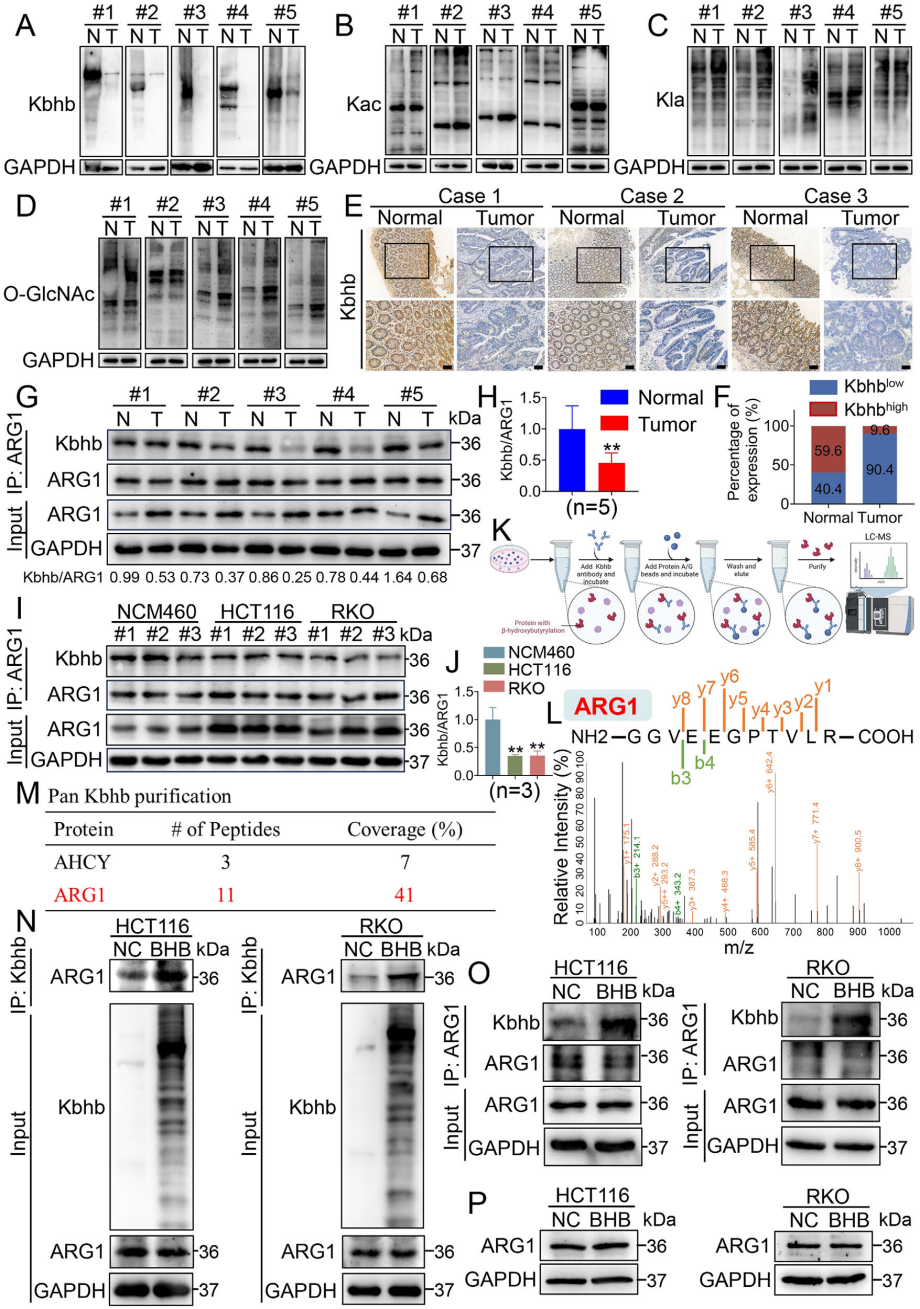
ARG1 is lowly β‐hydroxybutyrylated in CRC. A–D) Western blot analysis of lysine β‐hydroxybutyrylation (Kbhb) (A), acetylation (Kac) (B), lactation (Kla) (C), and O‐linked beta‐N‐acetylglucosamine (O‐GlcNAc) (D) levels in 5 matched CRC tissues (T) and adjacent noncancerous tissues (N). E) Three representative cases showed low levels of Kbhb in human primary CRC tissues and high levels of Kbhb in adjacent normal tissues analyzed by IHC staining. Scale bar: 50 µm. F) Quantitative analysis of Kbhb expression in paired CRC tissues. n=52. G) The expression of ARG1 Kbhb in 5 pairs of primary CRC tissues and matched adjacent noncancerous colon tissues. Semi‐quantification of proteins by scanning grayscale values using Image J. The ratio of the gray value of Kbhb to the ARG1 gray value was defined as the relative gray value of the modification. A low level of modification in CRC tissue was defined when the relative gray value of modification in adjacent normal tissue exceeded than that in CRC tissues, and vice versa. H) Quantitative analysis of Kbhb/ARG1 in paired CRC tissues. Data are presented as means ± SD; n=5. ^**^ *p* < 0.01, versus the Normal group. I) Cell lysates of NCM460, HCT116, and RKO were immunoprecipitated with anti‐ARG1 antibody, followed by immunoblotting. J) Quantitative analysis of Kbhb/ARG1 in NCM460, HCT116, and RKO cells. Data are presented as means ± SD; n=3. ^**^ *p* < 0.01, versus the NCM460 group. K) The whole flow chart of total lysine β‐hydroxybutyrylation via LC‐MS in HCT116 cells. Created in BioRender. Lin, C. (2025) https://BioRender.com/di3s84l. L) Cell lysates extracted from HCT116 cells were subjected to immunoprecipitation with anti‐Kbhb antibody. LC‐MS was used to analyze the potential proteins with Kbhb modification. The “b” and “y” symbols indicated the MS‐identified fragment ions from the N‐termini (b) and C‐termini (y) of the peptides after fragmentation. The presented diagram provided the MS/MS spectra of the identified ARG1 peptides. M) Table summarizes the vital proteins identified by LC‐MS analysis. We marked the protein of interest in red font. N, O) HCT116 and RKO cells were treated with BHB for 48 h, and whole‐cell extracts were collected for immunoprecipitation with anti‐Kbhb (N) or anti‐ARG1 (O) antibody, followed by immunoblotting. n=3. P) Western blot analysis of ARG1 levels in HCT116 and RKO cells from each group. n=3.

To explore the underlying mechanisms by which ARG1 exerts its oncogenic role independently of its catalytic activity in CRC, we turned to PTMs. To investigate the role of protein lysine acylations in CRC, four types of lysine acylations were investigated by western blotting in clinical samples from CRC patients. A significant reduction of lysine Kbhb was observed in CRC tissues relative to matched adjacent normal tissues (**Figure** **4**A), while only a slight elevation of lysine acetylation (Kac) (Figure 4B), lactation (Kla) (Figure 4C), and O‐linked N‐acetylglucosamine (O‐GlcNAc) (Figure 4D) was detected. IHC staining in clinical samples from CRC patients further revealed that Kbhb levels were significantly lower in tumor tissues compared with corresponding normal tissues (Figure 4E,F; Figure S3A, Supporting Information). Notably, Kbhb emerged as a significant modification. We next investigate whether the Kbhb level of ARG1 is altered in CRC patients. To conduct a more focused analysis, we selected 5 pairs of samples in which the difference in ARG1 expression between CRC tissues and matched adjacent normal tissues was less than ± 2‐fold to detect the levels of ARG1 Kbhb by immunoprecipitation. We found that ARG1‐Kbhb levels decreased in CRC tissue compared with those in paired noncancerous colorectal tissues (Figure 4G,H). We further validated it in CRC cell lines (HCT116 and RKO) and normal human colon epithelial cell line (NCM460). Consistent with our previous observations, ARG1 protein levels were increased in CRC cell lines (HCT116 and RKO) compared with those in normal human colon epithelial cell line (NCM460). However, we observed an obvious decrease in ARG1 Kbhb level in CRC cell lines compared with that in normal human colon epithelial cell line (Figure 4I,J). These data suggested that Kbhb and ARG1 Kbhb levels decreased in CRC. Therefore, we speculated that the oncogenic effect of ARG1 is mainly due to its low levels of Kbhb, even though its protein was highly expressed. Kbhb is an emerging PTM induced by BHB. Furthermore, BHB has been reported to induce acetylation. We found that BHB increased the levels of Kbhb but not the levels of Kac (Figure S3B,C, Supporting Information). To further explore whether BHB can induce the Kbhb of ARG1, we used a specific anti‐Kbhb antibody to pull down the potential proteins undergoing Kbhb modification in BHB‐treated HCT116 cells and then utilized a liquid chromatograph mass spectrometer (LC‐MS) to identify these proteins (Figure 4K), and coomassie blue staining was conducted (Figure S3D, Supporting Information). The LC‐MS analysis results showed that 41% of the amino acid sequence of ARG1 was successfully detected and identified, suggesting that ARG1 potentially harbors Kbhb modification in the presence of BHB (Figure 4L,M). Furthermore, the cell lysates from BHB‐treated or untreated cells were immunoprecipitated with anti‐Kbhb antibody and then immunoblotted with anti‐ARG1 antibody, or immunoprecipitated with anti‐ARG1 antibody and then immunoblotted with anti‐Kbhb antibody. The results showed that the level of ARG1 Kbhb from BHB‐treated cells was significantly higher than that from untreated cells, suggesting that Kbhb of endogenous ARG1 occurs via BHB (Figure 4N,O), whereas BHB failed to regulate the protein level of ARG1 (Figure 4P). Taken together, these data suggested that ARG1 is lowly β‐hydroxybutyrylated in CRC and BHB stimulates the Kbhb of ARG1.

**Figure 5:**
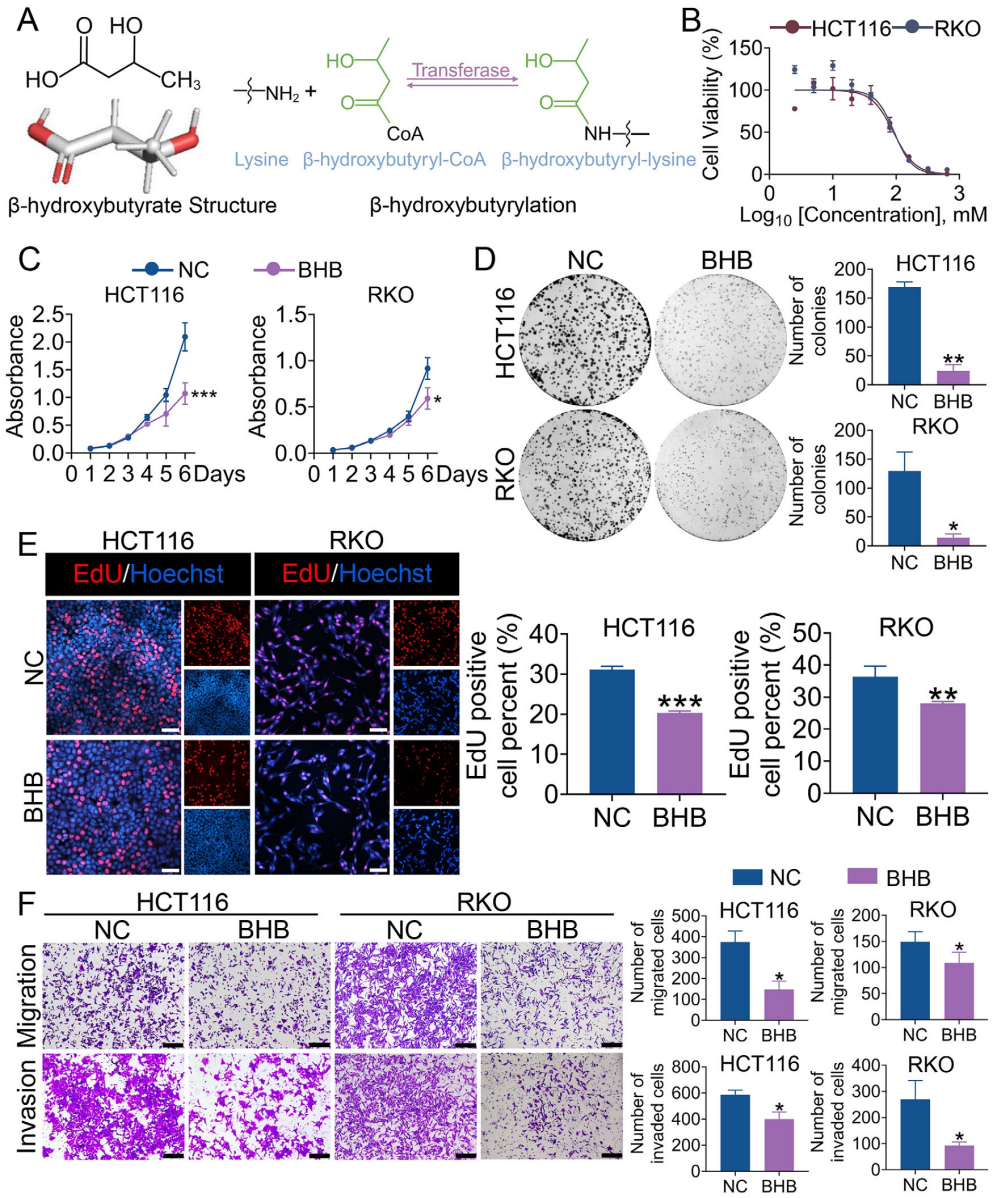
Kbhb of ARG1 induced by BHB inhibits the cell growth of CRC. A) Chemical structure of BHB and illustration for β‐hydroxybutyryllysine. Created in BioRender. Lin, C. (2025). B) Growth curves of HCT116 and RKO cells with different concentrations of BHB treatment. C, D) CCK‐8 assays (C) and colony formation assays (D) were performed in HCT116 and RKO cells treated with or without BHB. The right panel showed the statistics. E) EdU assays for HCT116 and RKO cells treated with or without BHB. The right panel showed the statistics. Scale bar: 50 µm. F) Transwell migration and invasion assays were performed in HCT116 and RKO cells treated with or without BHB. The right panel showed the statistics. Scale bar: 100 µm. Data are presented as means ± SD; n=3. ^*^ *p* < 0.05, ^**^ *p* < 0.01, ^***^ *p* < 0.001, versus the NC group.

To further determine the responses of CRC cells to ARG1 Kbhb, we explored the potential role of BHB in CRC (**Figure** **5**A). According to the results, BHB demonstrated the IC50 values at 91.22 mmol L^−1^ in HCT116 cells and 93.64 mmol L^−1^ in RKO cells (Figure 5B). Therefore, 30 mmol L^−1^ BHB was selected for further validation. CRC cells treated with BHB exhibited decreased cell viability, proliferation ability, and colony formation ability compared with the normal control cells (Figure 5C–E). Moreover, treatment with BHB diminished the migration and invasion of HCT116 and RKO cell lines (Figure 5F). Taken together, these data suggested that treatment with BHB inhibited the proliferation, migration, and invasion of CRC in vitro.

### 2.5 P300 Catalyzes ARG1 Kbhb and Stimulates ARG1 Interacting with SLC3A2

**Figure 6:**
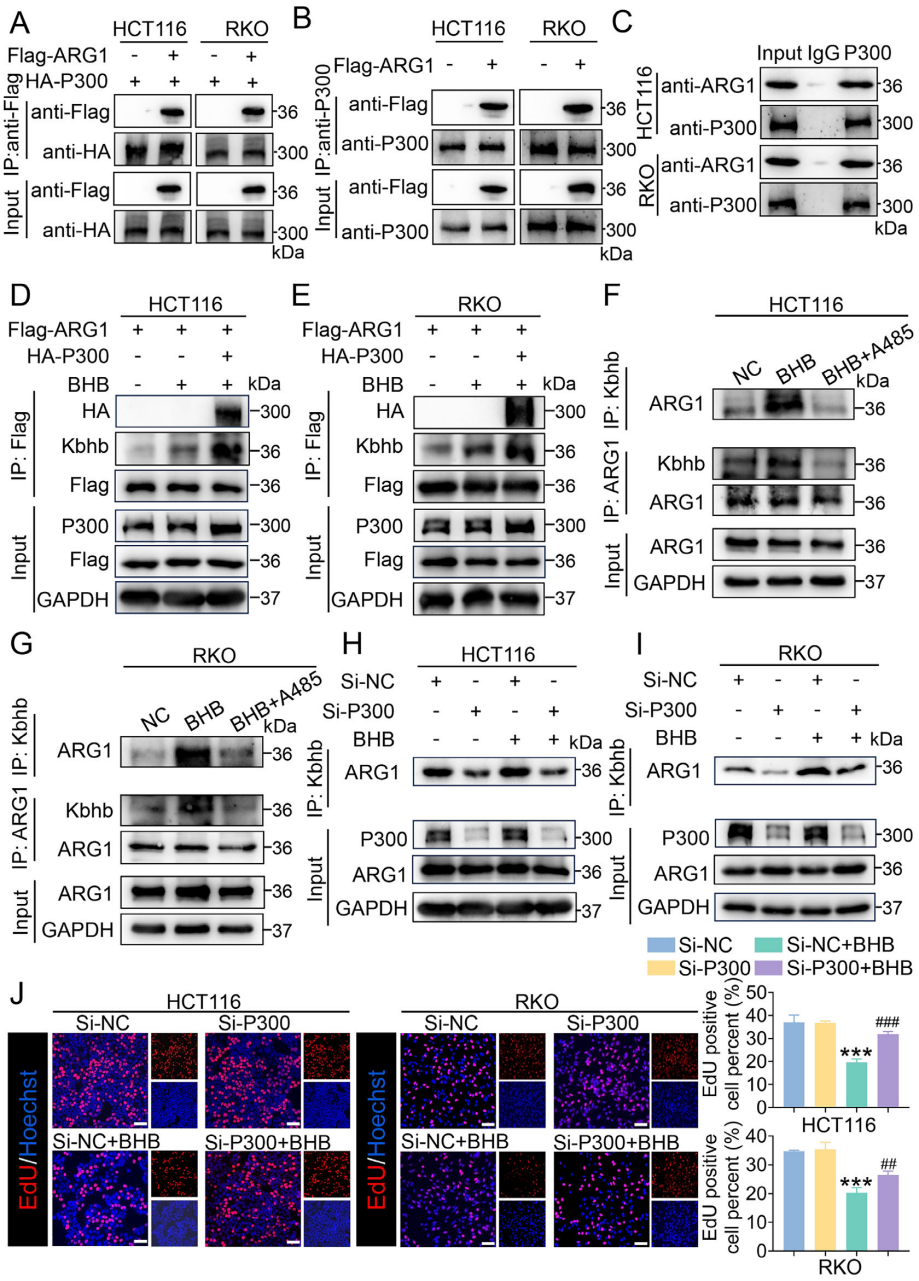
P300 Catalyzes the Kbhb of ARG1. A–C) Exogenous (A), semi‐exogenous (B), and endogenous (C) Co‐IP were performed to detect the interaction between ARG1 and P300 in HCT116 and RKO cells. D, E) HCT116 (D) and RKO (E) cells were transfected with Flag‐ARG1 or co‐transfected with Flag‐ARG1 and HA‐P300, then treated with or without BHB, and then subjected to precipitation with Flag beads, followed by immunoblotting. F, G) A485 reduced the Kbhb level of ARG1. Cell lysates in indicated groups of HCT116 (F) and RKO (G) cells were immunoprecipitated with anti‐Kbhb or anti‐ARG1 antibody, followed by immunoblotting. H, I) Silencing P300 reduced the Kbhb level of ARG1 in HCT116 (H) and RKO (I) cells. Cells were transfected with siRNA oligos specially targeting P300 or a negative control, followed by treating with or without BHB after 48 h. J) EdU assays in ARG1 knockdown HCT116 and RKO cells. The right panel showed the statistics. Scale bar: 50 µm. Data are presented as means ± SD; n=3. ^***^ *p* < 0.001, versus the Si‐NC group. ^##^ *p* < 0.01, ^###^ *p* < 0.001, versus the Si‐P300+BHB group.

P300 has been identified as an acyltransferase for acetylation, propionylation, and crotonylation.^[^ ^27^ ^]^ Recent studies showed that it could catalyze the Kbhb reaction^[^ ^23^ ^]^ (Figure S4A, Supporting Information). First, we detected the expression level of P300 in CRC cell lines treated with or without BHB. The results showed BHB failed to regulate the expression level of P300 (Figure S4B, Supporting Information), suggesting that BHB promotes Kbhb levels by providing Kbhb donors to enhance intracellular Kbhb levels rather than by affecting P300 expression. We next identified whether P300 catalyzed the Kbhb of ARG1. The exogenous, semi‐exogenous, and endogenous Co‐IP assays showed that ARG1 can interact with P300 (**Figure** **6**A–C). Consistent with the expected results, the P300 over‐expression led to an increase in the Kbhb levels of ARG1 (Figure 6D,E). To further investigate whether P300 was involved in Kbhb of ARG1, HCT116 and RKO cells were treated with BHB or with P300 inhibitor A485 (Figure S4C, Supporting Information). Immunoblot results showed that BHB increased the levels of Kbhb in CRC cell lines while A485 inhibited the levels of Kbhb (Figure S4D,E, Supporting Information). Besides, Co‐IP assays indicated that P300 inhibitor A485 inhibited the level of ARG1 Kbhb (Figure 6F,G). Analogously, depletion of P300 by siRNA also reduced the Kbhb of ARG1 induced by BHB (Figure 6H,I). The results demonstrated that P300 can act as a β‐hydroxybutyryl‐transferase for ARG1. Along with the reduced ARG1 Kbhb level, depletion of P300 significantly reversed the reduction in proliferation ability induced by BHB in cells treated with si‐P300 and BHB. Importantly, depletion of P300 did not dramatically alter proliferation ability in the absence of BHB, indicating that P300 exerted its functions via promoting Kbhb (Figure 6J).

**Figure 7:**
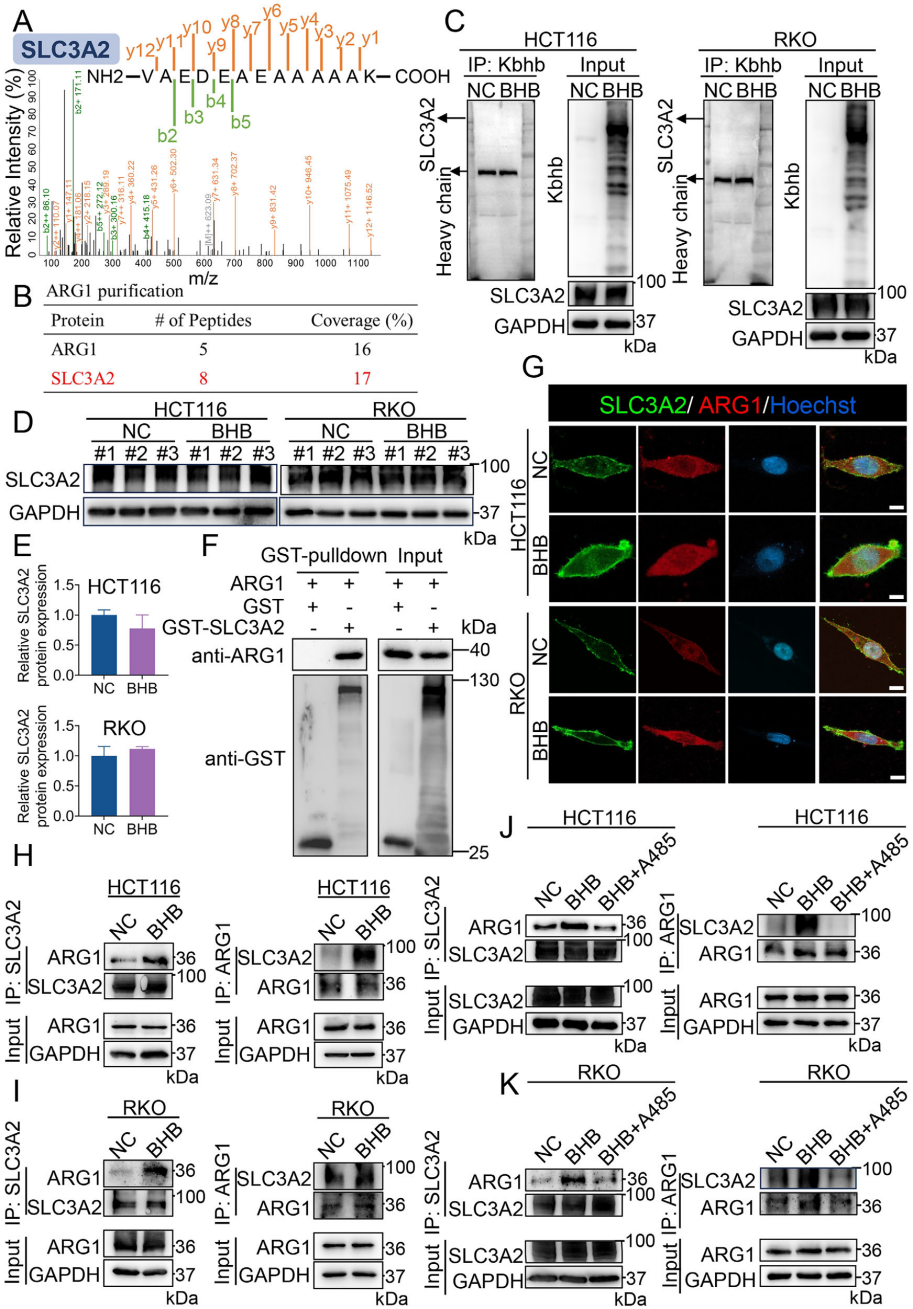
The Kbhb of ARG1 stimulates the interaction between ARG1 and SLC3A2. A) Cell lysates extracted from HCT116 cells were subjected to immunoprecipitation with anti‐ARG1 antibody. LC‐MS was used to analyze the potential proteins interacting with ARG1. The “b” and “y” symbols indicated the MS‐identified fragment ions from the N‐termini (b) and C‐termini (y) of the peptides after fragmentation. The presented diagram provided the MS/MS spectra of the identified SLC3A2 peptides. B) The table summarizes the vital proteins identified by LC‐MS analysis. We marked the protein of interest in red font. C) Immunoprecipitation and western blot analysis of indicated targets in HCT116 and RKO cells treated with or without BHB. D) Western blot analysis of SLC3A2 levels in HCT116 and RKO cells treated with or without BHB. E) Relative protein expression of SLC3A2 in HCT116 and RKO cells. F) Direct binding of ARG1 to SLC3A2 using the GST pull‐down assay. G) Immunofluorescence analysis of SLC3A2 and ARG1 protein in HCT116 and RKO cells treated with or without BHB. Scale bar: 10 µm. H, I) Cell lysates in indicated groups of HCT116 (H) and RKO (I) cells were immunoprecipitated with anti‐SLC3A2 or anti‐ARG1 antibody, followed by immunoblotting. J, K) HCT116 (J) and RKO (K) cells were treated with BHB for 48 h, and whole‐cell extracts were collected for immunoprecipitation with anti‐SLC3A2 or anti‐ARG1 antibody, followed by immunoblotting. Data are presented as means ± SD; n=3.

To further explore how ARG1 Kbhb regulated CRC progression, we searched for ARG1 Kbhb‐interacting proteins by immunoprecipitation with anti‐ARG1 antibodies in HCT116 cells treated with BHB. LC‐MS analysis showed that ARG1 Kbhb may interact with solute carrier family 3 member 2 (SLC3A2) (**Figure** **7**A,B; Figure S5A, Supporting Information). Besides, BHB failed to regulate the level of SLC3A2 and Kbhb of SLC3A2 (Figure 7C–E). To further investigate whether ARG1 binds to SLC3A2 directly, we purified human SLC3A2 protein with a glutathione‐S‐transferase (GST) tag. Using purified proteins of GST‐SLC3A2, we performed a GST beads pull‐down assay and found that ARG1 bound to SLC3A2 directly, but not GST (Figure 7F). An IF assay was performed to confirm the co‐localization of ARG1 and SLC3A2. The results showed that ARG1 was distributed in both the cell membrane and cytoplasm, while SLC3A2 was predominantly located in the cell membrane of CRC cells. Besides, IF results confirmed that BHB promoted the interaction between ARG1 and SLC3A2 in HCT116 and RKO cells (Figure 7G). Exogenous Co‐IP assays in HCT116 and RKO cells further confirmed BHB promoted the interaction between ARG1 and SLC3A2 (Figure 7H,I). Collectively, these data revealed that the Kbhb of ARG1 enhanced its interaction with SLC3A2. We next investigated whether P300‐mediated Kbhb of ARG1 regulates the interaction between ARG1 and SLC3A2. The results showed that A485 dramatically diminished the formation of ARG1 Kbhb/SLC3A2 complex in CRC cells (Figure 7J,K). The findings indicated P300 exhibited Kbhb transferase activity to mediate Kbhb of ARG1 and stimulated ARG1 interacting with SLC3A2.

### 2.6 ARG1 Kbhb Leads to the Reprogramming of Arginine Metabolism by Interacting with SLC3A2 in CRC

**Figure 8:**
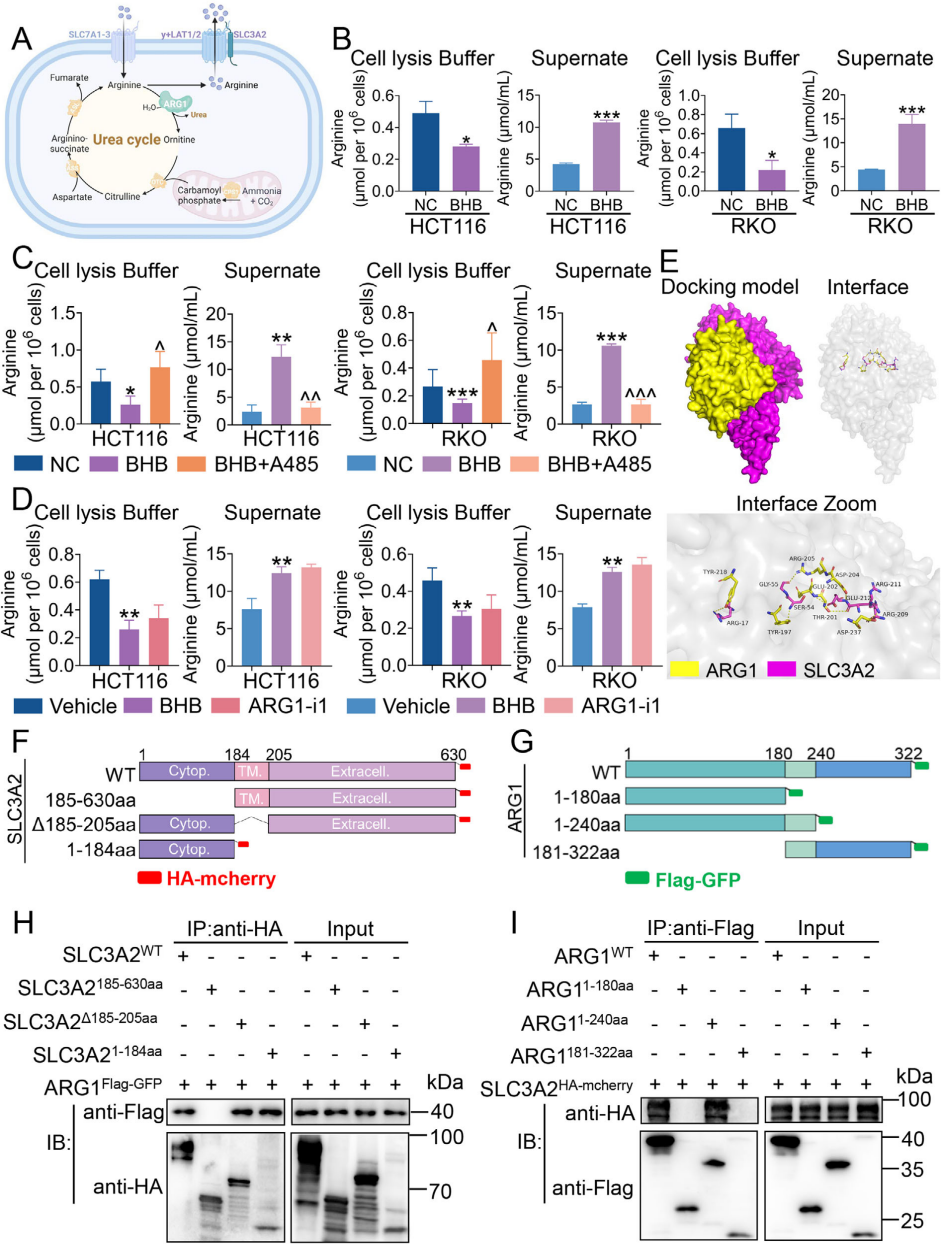
ARG1 Kbhb leads to the reprogramming of arginine metabolism by interacting with SLC3A2 in CRC. A) Illustration depicting the process of arginine metabolism in CRC cells. Created in BioRender. Lin, C. (2025) https://BioRender.com/ukdx9zx. B) The intracellular and extracellular arginine content in HCT116 and RKO cells treated with or without BHB, respectively. C) The intracellular and extracellular arginine content in HCT116 and RKO cells treated with or without BHB or BHB+A485, respectively. D) The intracellular and extracellular arginine content in HCT116 and RKO cells treated with DMSO or BHB, or ARG1‐i1, respectively. E) Surface diagram of the docking model and their interfacing residues between ARG1 and SLC3A2 protein (ARG1, yellow; SLC3A2, rose red; hydrogen bond interaction, dotted line). F, G) Schematic illustration of SLC3A2 truncated constructs (F) and ARG1 truncated constructs (G). H) Co‐IP analysis on association of HA‐tagged SLC3A2 (WT), SLC3A2 (T+E), SLC3A2 (C+E), and SLC3A2 (C) with Flag‐tagged ARG1 in HEK 293 T cells. I) Co‐IP analysis on association of Flag‐tagged ARG1^WT^, ARG1^181‐322aa^, ARG1^1‐240aa,^ and ARG1^1‐180aa^ with HA‐tagged SLC3A2 in HEK 293 T cells. Data are presented as means ± SD; n=3. ^*^ *p* < 0.05, ^**^ *p* < 0.01, ^***^ *p* < 0.001, versus the NC or Vehicle group; ^*p* < 0.05, ^^*p* < 0.01, ^^^*p* < 0.001, versus the BHB group.

Accumulating evidence has demonstrated that SLC3A2 (CD98hc)‐y^+^LAT1 (SLC7A7) and SLC3A2‐y^+^LAT2 (SLC7A6) heterodimers play a role in the efflux of cationic amino acids, such as arginine, lysine, and ornithine in exchange for neutral amino acids and Na^+[^ ^28^, ^29^ ^]^(**Figure** **8**A). We have previously demonstrated the Kbhb of ARG1 enhanced its interaction with SLC3A2. Next, we investigated whether the ARG1 Kbhb/SLC3A2 complex regulated the arginine metabolism, which was required for tumorigenicity. First, we observed that BHB treatment in CRC cell lines significantly reduced the levels of arginine in intracellular and increased the levels of arginine in extracellular compared to control CRC cells (Figure 8B). Furthermore, inhibiting ARG1 Kbhb by A485 resulted in an increase in intracellular arginine levels and a decrease in extracellular arginine levels in CRC cells, indicating the Kbhb of ARG1 regulated intracellular and extracellular arginine metabolism by affecting ARG1 Kbhb/SLC3A2 complex (Figure 8C). However, inhibition of ARG1 enzymatic activity by ARG1‐i1 failed to regulate the intracellular and extracellular arginine levels, suggesting ARG1 Kbhb regulated the intracellular and extracellular arginine levels independently of its catalytic activity (Figure 8D). To verify the relationship between ARG1 and SLC3A2, we performed rigid protein‐protein docking (Figure 8E). ARG1 and SLC3A2 formed bonds through amino acid residue sites, revealing that ARG1 and SLC3A2 formed a stable protein docking model. To further identify the binding domain of ARG1 that interacts with SLC3A2, we constructed truncated HA‐tagged SLC3A2 and truncated Flag‐tagged ARG1 plasmids based on the docking site (Figure 8F,G). We constructed full‐length ARG1 and truncated HA‐tagged SLC3A2. The results revealed that the intracellular domain of SLC3A2 directly interacted with ARG1 (Figure 8H), which was further confirmed by GST pull‐down assay (Figure S6A, Supporting Information). Then we co‐transfected with SLC3A2^WT^ and truncated Flag‐tagged ARG1 plasmid into HEK 293 T cells to perform a Co‐IP assay. The result showed that the 1‐240 amino acid of ARG1 was crucial for the interaction with SLC3A2 (Figure 8I). These findings indicated that the Kbhb of ARG1 promoted the efflux of arginine via interacting with the cytoplasmic domain of SLC3A2, underscoring the pivotal role of ARG1 Kbhb in arginine metabolic reprogramming.

### 2.7 Kbhb of ARG1 at Lys313 Affects Its Binding to SLC3A2

**Figure 9:**
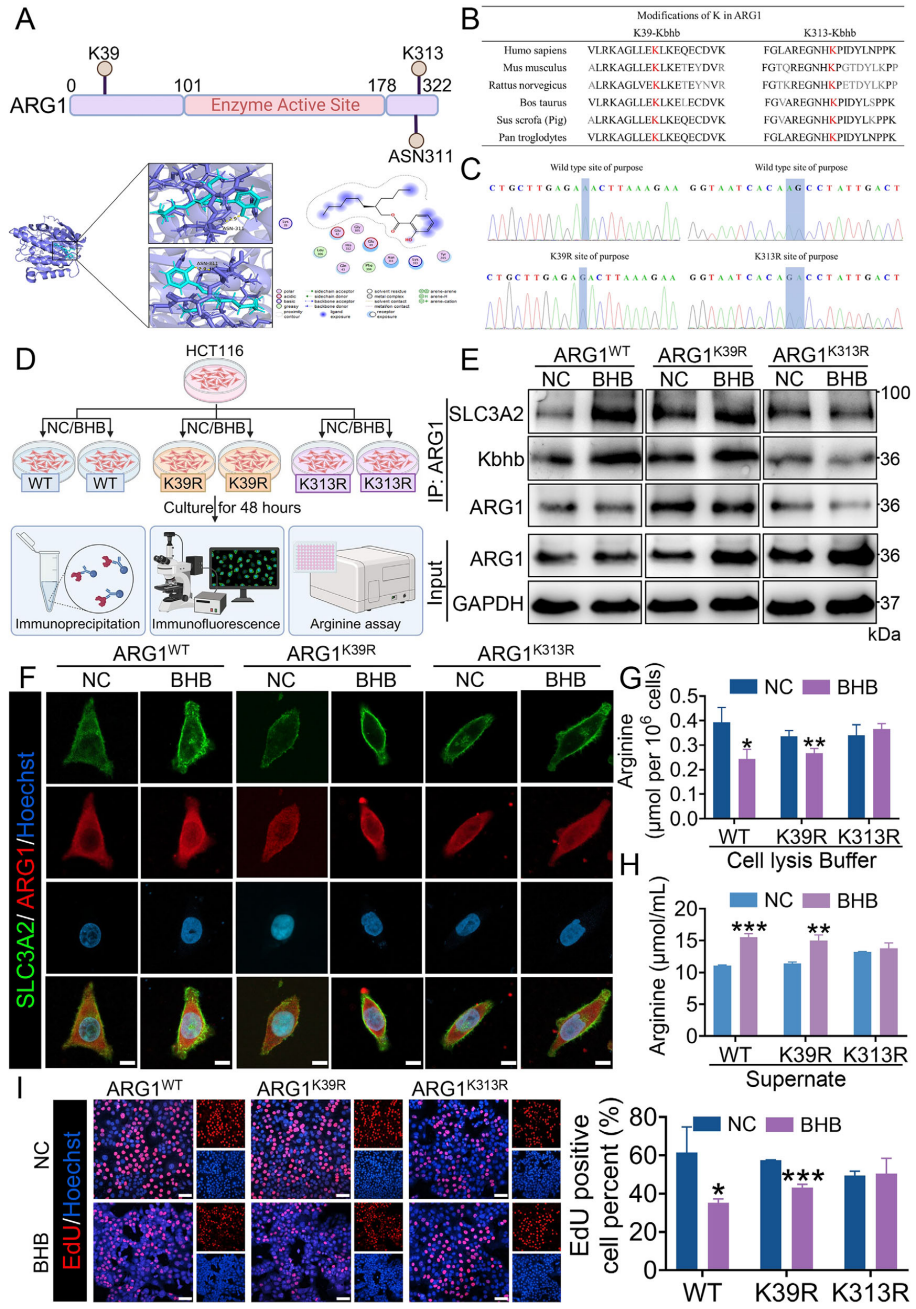
Kbhb of ARG1 at Lys313 affects its binding to SLC3A2, regulates arginine metabolism, and inhibits cell proliferation. A) Molecular docking for the prediction of the binding affinity between the ARG1 and BHB molecule. Created in BioRender. Lin, C. (2025) https://BioRender.com/l1sccv7. B) ARG1‐K39 and ‐K313 sequence alignment from the indicated species is shown. C) Construction of ARG1^WT^, ARG1^K39R,^ and ARG1^K313R^ plasmids, and validated them by OBiO Technology Sequencing. D) Schematic of ARG1‐WT or ‐K39R or ‐K313R HCT116 cells treated with or without BHB, followed by immunoprecipitation, immunofluorescence, and arginine assay. Created in BioRender. Lin, C. (2025) https://BioRender.com/fxzd0hh. E) Flag‐ARG1^WT^, Flag‐ARG1^K39R,^ and Flag‐ARG1^K313R^ were transfected into HCT116 cells for 24 h, then treated with BHB for 48 h. Lysates were immunoprecipitated with anti‐ARG1 antibody, followed by Western blot for target identification. F) Immunofluorescence analysis of SLC3A2 and ARG1 protein in ARG1‐WT or ‐K39R or ‐K313R HCT116 cells treated with or without BHB. Scale bar: 10 µm. G, H) The intracellular (G) and extracellular (H) arginine content in ARG1‐WT or ‐K39R or ‐K313R HCT116 cells treated with or without BHB, respectively. I) EdU assays for ARG1‐WT or ‐K39R or ‐K313R HCT116 cells treated with or without BHB. The right panel showed the statistics. Scale bar: 50 µm. Data are presented as means ± SD; n=3. ^*^ *p* < 0.05, ^**^ *p* < 0.01, ^***^ *p* < 0.001, versus the NC group.

Next, we sought to identify the Kbhb sites on ARG1. Molecular docking in the Molecular Operating Environment (MOE) was employed to predict the binding affinity between β‐hydroxybutyrate molecules and ARG1 protein, consistent with the method reported previously,^[^ ^30^, ^31^ ^]^ and the results indicated that two lysine sites (Lys39 and Lys313 of ARG1) were predicted to be potentially β‐hydroxybutyrylated (**Figure** **9**A). Notably, Lys39 and Lys313 are highly conserved amino acid residue in ARG1 across various species (Figure 9B). Thus, we mutated these two potential Kbhb sites by substituting lysine (K) with arginine (R) to explore novel modes of ARG1 regulation via Kbhb (Figure 9C,D). Western blot after anti‐ARG1 immunoprecipitation confirmed direct Kbhb of the K313 site within the ARG1 protein but not the K39 site (Figure 9E). Subsequently, we investigated whether direct Kbhb of ARG1 affected its ability to bind to SLC3A2. Co‐IP assay showed that the SLC3A2 bound by ARG1^WT^ and ARG1^K39R^ were more abundant than those bound by ARG1^K313R^ (Figure 9E). Consistently, IF assay showed no alternation in the subcellular co‐localization of ARG1^K313R^ and SLC3A2, indicating that K313 was a key Kbhb site on ARG1 (Figure 9F). To gain deeper insights into the function of ARG1 Kbhb on the K313 site, we detected the levels of arginine in intracellular and extracellular. Significantly, the levels of arginine were decreased in intracellular and increased in extracellular of HCT116 cells transfected with *ARG1* ^WT^ or *ARG1* ^K39R^ in the BHB treatment group compared to the NC treatment group, whereas no alternation of arginine levels in intracellular and extracellular transfected with *ARG1* ^K313R^ between NC and BHB group (Figure 9G,H). We then examined the impact of *ARG1* ^K39R^/*ARG1* ^K313R^ on the cell proliferation of HCT116 cells. The results showed that *ARG1* ^WT^/*ARG* ^K39R^ in the BHB treatment group significantly decreased proliferation ability compared to the NC treatment group, while there was no difference in cells transfected with the *ARG1* ^K313R^ between the NC and BHB group, indicating that the Kbhb of ARG1 at Lys313 played a crucial role in cell proliferation (Figure 9I). Therefore, our data conclusively demonstrated that BHB facilitated the direct Kbhb of ARG1 at the Lys313 site, enhancing its SLC3A2‐binding capacity and thereby regulating the arginine metabolism‐mediated progression of CRC.

### 2.8 The Combination of BHB with ARG1 Inhibitor Inhibits the Growth of CRC

**Figure 10:**
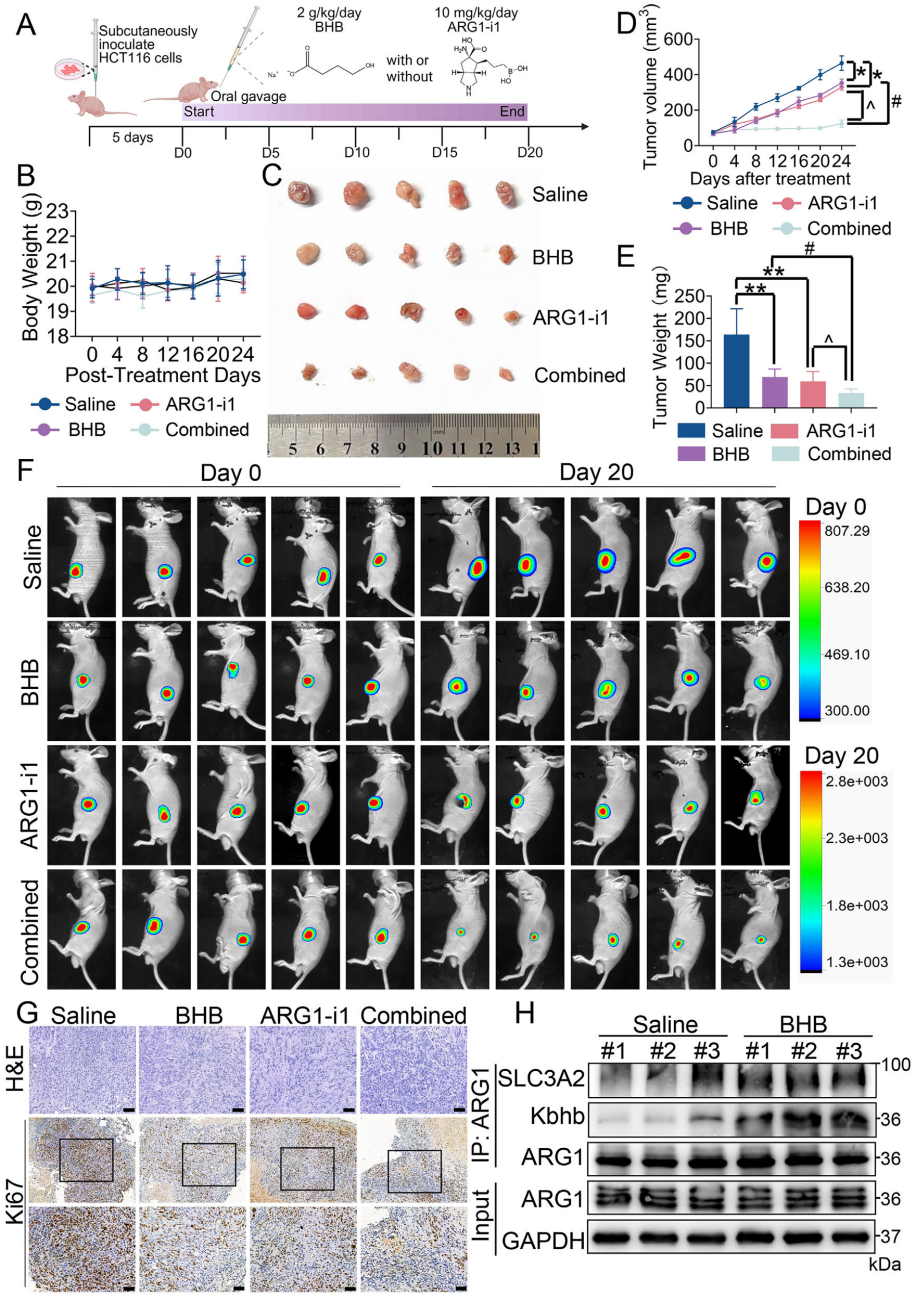
Identification of an efficacious therapy of ARG1 inhibitor in combination with BHB in CRC. A) Schematic diagram showing the combined therapy effect of BHB and ARG1‐i1 in BALB/c nude mice. Created in BioRender. Lin, C. (2025) https://BioRender.com/v73953i. B) Body weights of mice from each group were measured. C) Images of the dissected subcutaneous tumors from the tumor‐bearing mice at the end of the xenograft BALB/c nude mice model (n = 5 mice per group). D) Time‐course evaluation of subcutaneous tumor volume measured every 4 days in BALB/c nude mice. E) The final weight of the subcutaneous tumors was shown in the histogram for the indicated groups. F) In vivo bioluminescent images of subcutaneous tumor formed by subcutaneous injection of HCT116 cells in BALB/c nude mice (n = 5 in each group), administered saline or BHB or ARG1‐i1, or BHB and ARG1‐i1 through daily gavage administration. G) Representative images of HE and Ki67 IHC staining in different treatment groups. Scale bar: 50 µm. H) Tumor tissue lysates of the Saline group and BHB group were immunoprecipitated with anti‐ARG1 antibody, followed by immunoblotting. Data are presented as means ± SD; n=5. ^*^ *p* < 0.05, ^**^ *p* < 0.01, versus the Saline group; #*p* < 0.05, versus the BHB group, ^*p*< 0.05, versus the ARG1‐i1 group.

We next performed a xenograft‐induced model, in which mice were administered BHB or ARG1‐i1 or co‐administered BHB and ARG1‐i1 (**Figure** **10**A). To demonstrate the safety and applicability of BHB and ARG1‐i1, in vivo experiments were conducted in mice to evaluate their systemic toxicity and vital organ toxicity. Daily treatment of BALB/c nude mice with BHB or ARG1‐i1 did not result in any significant changes in body weight (Figure 10B) or in the histopathological analyses of major organs, including the heart, kidney, liver, lung, and spleen (Figure S7A, Supporting Information), when compared with those of the Saline group. This indicated that co‐administration of BHB with ARG1‐i1 did not cause obvious toxicity in vivo. We then evaluated the therapeutic efficacy of BHB and ARG1‐i1. The results showed that treatment with either compound alone effectively reduced subcutaneous tumor growth. Moreover, administration of BHB with ARG1‐i1 in combination demonstrated a significantly enhanced inhibitory effect on tumor growth compared with individual treatments (Figure 10C–E). The results were also confirmed by in vivo bioluminescent analysis (Figure 10F). Furthermore, Ki67, a marker indicative of cell proliferation, showed significant downregulation in proliferative activity in the BHB group and the ARG1‐i1 group, especially in co‐administration of BHB with the ARG1‐i1 group compared to the Saline group (Figure 10G). Consistent with the aforementioned findings, Co‐IP assays in mouse tumor tissue confirmed notable increase of ARG1 Kbhb and its binding to SLC3A2 following BHB treatment (Figure 10H). Besides, consistent with the results in vitro, no changes in the expression of ARG1 and SLC3A2 were observed in mouse tumor tissue even after long‐term treatment with BHB and ARG1‐i1 (Figure S7B,C, Supporting Information). Overall, these findings showcased that ARG1 acted as a promising target for PTM regulation in combination with its enzymatic activity blockade.

## 3 Discussion

Metabolic reprogramming is a hallmark of malignancy.^[^ ^32^ ^]^ Alterations in arginine metabolism play a critical role in tumor development and progression.^[^ ^33^, ^34^, ^35^ ^]^ Emerging evidence indicated that elevated arginine levels were necessary for tumor progression, including HCC, prostate cancer, breast cancer, and leiomyosarcoma.^[^ ^11^, ^12^, ^13^, ^36^ ^]^ In the present study, we found that the arginine metabolism pathway was enriched in CRC. Besides, arginine levels in CRC tissues were significantly increased compared with paired normal mucosa tissues. As a critical catalytic enzyme in arginine metabolism, ARG1 participates in cancer development through diverse mechanisms. The inhibition of ARG1 and the resulting decrease in arginine catabolism to ornithine caused an imbalance of amino acid metabolites, particularly the reduced synthesis of polyamines from arginine. Polyamine metabolites are associated with diverse biological processes such as proliferation, apoptosis, and cellular stress responses.^[^ ^33^, ^37^, ^38^ ^]^ Apart from its critical catalytic enzymic function in the urea cycle for the removal of toxic ammonia and synthesis of polyamines, it also mediated a variety of cellular functions and processes such as proliferation, senescence, apoptosis, autophagy as well as inflammatory responses in various types of cells in an arginase activity‐dependent or independent manner.^[^ ^37^, ^39^, ^40^, ^41^, ^42^ ^]^ Besides, studies have shown that tumor cells consume arginine in the tumor microenvironment through high expression of ARG1, thereby inhibiting immune T cell activation and driving immune suppression in cancer.^[^ ^43^ ^]^ Recently, the basic and clinical studies suggested ARG1 is also highly expressed in various cancers such as breast cancer, gastric cancer, CRC, and HCC.^[^ ^44^, ^45^, ^46^, ^47^, ^48^ ^]^ In this study, scRNA‐seq analysis from the database revealed that ARG1 was highly expressed in CRC tissues. To verify these findings, western blot and IHC staining demonstrated that ARG1 was significantly upregulated in CRC tissues. The OS analysis of patients with CRC suggested that ARG1 was a sensitive predictor of poor prognosis. However, we found that there was no correlation between ARG1 expression and pathological characteristics, including TNM stage, lymph node metastasis, and vessel invasio, which may be due to the small sample size of this study (n = 53). Taken together, these results demonstrated the crucial role of ARG1 in CRC development; however, further elucidation of its specific mechanism in cancer progression was needed.

Our investigations initially focused on the well‐established role of ARG1 expression levels in CRC progression. However, emerging evidence in the field of epigenetics has directed our attention to PTMs as key regulators of protein function. The protein PTMs are key steps in the process of regulating cellular functions and activities, including, but not limited to, metabolic reprogramming, protein stability and transport, and transcriptional regulation.^[^ ^49^, ^50^, ^51^, ^52^, ^53^ ^]^ Notably, protein Kbhb on lysine is a recently discovered modification type, and Kbhb is a promising therapeutic target for cancer treatment.^[^ ^25^, ^26^, ^54^ ^]^ Western blot and IHC analysis revealed a significant reduction in overall Kbhb levels in primary CRC tissues compared with matched noncancerous colorectal samples. This finding led us to further interrogate the status of Kbhb specifically on ARG1. A previous MS analysis of the β‐hydroxybutyrylome in mouse liver revealed 891 sites of Kbhb within 267 proteins enriched for fatty acid, amino acid, detoxification, and one‐carbon metabolic pathways. Among them, Kbhb of S‐adenosyl‐L‐homocysteine hydrolase (AHCY) inhibited its enzymatic activity, which might be a possible therapeutic target in cancer. In addition, this MS analysis also suggested that ARG1 may be β‐hydroxybutyrylated.^[^ ^21^ ^]^ Therefore, further research on ARG1 Kbhb may significantly improve innovation in cancer therapy. In this study, we presented the elevated expression levels of ARG1 but low levels of Kbhb in ARG1 in CRC. LC‐MS analysis further confirmed the Kbhb of ARG1 induced by BHB, which led to the inhibition of cell proliferation and metastasis in a catalytic‐activity‐independent manner. Collectively, our results implied that although the level of ARG1 protein level was elevated, the abnormal Kbhb modification of ARG1 is a key target that facilitates CRC progression.

To explore the underlying mechanisms by which ARG1 Kbhb regulated the progression of CRC, LC‐MS analysis was conducted. We found that ARG1 was significantly β‐hydroxybutyrylated in CRC induced by BHB, and identified SLC3A2 as the downstream target of ARG1 in a Kbhb‐dependent manner. SLC3A2, a type II transmembrane glycoprotein, also known as CD98hc, serves as a chaperone for LAT1 (SLC7A5), LAT2 (SLC7A8), y^+^LAT1 (SLC7A7), y^+^LAT2 (SLC7A6), xCT (SLC7A11) and Asc1 (SLC7A10) providing their recruitment to the plasma membrane.^[^ ^55^, ^56^, ^57^, ^58^ ^]^ Typically, SLC3A2 ‐y^+^LAT1 and SLC3A2 ‐y^+^LAT2 heterodimers play a role in the efflux of cationic amino acids, such as arginine, lysine, and ornithine, in exchange for neutral amino acids and Na^+^ in intestinal epithelial cells.^[^ ^28^, ^29^, ^59^ ^]^ Here, we found that the promotion of ARG1 Kbhb through BHB significantly reduced intracellular arginine levels and increased extracellular arginine levels. Molecular docking revealed that ARG1 and SLC3A2 formed a stable protein docking model, and the intracellular domain of SLC3A2 directly interacting with ARG1 was further confirmed. These findings indicated that the Kbhb of ARG1 promoted the efflux of arginine via interacting with the cytoplasmic domain of SLC3A2, leading to a decrease in intracellular arginine levels and ultimately inhibiting CRC progression. The elevated arginine levels in tumor cells caused increased polyamine synthesis through arginine catabolism to ornithine, promoting tumorigenicity.^[^ ^60^ ^]^ Besides, high levels of arginine in breast cancer cells drove tumor‐associated macrophages (TAMs) to polarize into tumor‐promoting phenotype by secreting into TME, thereby inhibiting CD8^+^ T cell activity.^[^ ^61^ ^]^ The high levels of arginine in HCC also promoted tumor formation via binding RNA‐binding motif protein 39 (RBM39) to control expression of metabolic genes, including changes in glucose, amino acid, nucleotide, and fatty acid metabolism.^[^ ^36^ ^]^ Arginine can also be interconverted with proline and glutamate and can facilitate cell growth by triggering mTORC1 activation.^[^ ^9^ ^]^ Therefore, BHB‐induced upregulation of ARG1 Kbhb reduced intracellular arginine levels, likely via the aforementioned mechanisms, and consequently alleviated CRC suppression. Taken together, these results underscore the pivotal role of ARG1 Kbhb/SALC3A2 in arginine metabolic reprogramming.

P300 acts as a Kbhb transferase and has corresponding enzymatic activity both in vitro and in vivo.^[^ ^23^ ^]^ Our study found that ARG1 interacted with P300. Besides, the P300 overexpression led to an increase in the Kbhb levels of ARG1. Depletion of P300 by siRNA or inhibition of P300 by A485, a recently reported potent P300 inhibitor,^[^ ^62^ ^]^ reduced both Kbhb levels on ARG1 and the interaction of ARG1 with SLC3A2. Further molecular docking showed that the pocket of P300 can accommodate BHB. Additionally, A485 treatment increased intracellular arginine levels and decreased extracellular arginine levels. In summary, these results indicated that P300 served as the Kbhb “writer” of ARG1, thereby regulating arginine metabolic reprogramming. Understanding the Kbhb sites on non‐histone proteins is crucial because they play a significant role in tumor progression. Then, single‐point mutation and immunoprecipitation assays demonstrated that K313, rather than K39, of ARG1 protein modified by BHB was essential for its interaction with SLC3A2, and this interaction was crucial for  regulating the efflux of arginine. Our experiments using truncated plasmids revealed that the 1‐240 amino acid region of ARG1 was critical for its interaction with SLC3A2. We proposed that the Kbhb modification at K313 may influence the spatial conformation of ARG1, thereby enhancing the binding of the 1‐240 amino acid region to SLC3A2. This could be due to PTMs causing local conformational changes that propagate through the structure of the protein, ultimately affecting the interaction interface.^[^ ^63^ ^]^ Furthermore, MOE indicated that there was a hydrogen bonding interaction at the ASN311 site in the molecular modeling of the BHB‐ARG1 binding site, while the Kbhb at the K313 residue may alter the surrounding electrostatic environment, further influencing protein conformation and interaction affinity. Herein, we uncovered an interesting mechanism in which BHB induced ARG1 Kbhb at Lys313 to promote the interaction of ARG1 with SLC3A2, resulting in the efflux of arginine in CRC cells. Hence, targeting ARG1 Kbhb may reprogram the arginine metabolism.

Recent studies have reported that arginase plays a significant role in cancer progression by promoting immunosuppression and tumor growth, and targeting arginase activity through inhibitors or arginine deprivation could be a promising strategy for cancer therapy.^[^ ^64^, ^65^, ^66^, ^67^ ^]^ To enhance the potential clinical applicability of this research, we used a small‐molecule inhibitor of ARG1 to block its enzymatic activity in combination with BHB to promote its Kbhb level. Hence, the CRC tumor xenograft mouse model was employed, and the encouraging results supported the potential ability of ARG1 for cancer therapy in CRC, encompassing its conventional enzymatic role and its nonenzymatic‐metabolic function. Besides, the in vivo result further confirmed the higher expression levels of ARG1‐Kbhb induced by BHB compared to those of mice administered with saline, respectively. Our work uncovered a previously uncharacterized and unexpected molecular mechanism as well as therapy potential of ARG1 in CRC, which strengthened our understanding of the essential nonenzymatic‐metabolic functions and novel molecular action of ARG1 interacting with SLC3A2 in arginine metabolism reprogramming for tumorigenesis.

An important limitation of our study was that ARG1 inhibitors had different effects in vitro and in vivo. This discrepancy may be due to 1) The tumor microenvironment (TME) regulation. In vitro, CRC cells were cultured in a single environment, while in vivo, ARG1 inhibitors could affect tumor progression by modulating the polarization of tumor‐associated macrophages and restoring L‐arginine levels in the TME.^[^ ^68^, ^69^, ^70^, ^71^, ^72^, ^73^ ^]^ 2) The interaction between the immune system and ARG1 inhibitor therapy in vivo. ARG1 inhibitors could indirectly suppress tumors by activating or enhancing the host immune system and modulating myeloid cell functions.^[^ ^69^, ^71^, ^74^, ^75^ ^]^ 3) Metabolic differences between in vitro and in vivo. The metabolic competition between tumor and immune cells was more pronounced in vivo, and ARG1 inhibitors may preferentially restore the metabolic needs of immune cells.^[^ ^71^, ^72^, ^76^, ^77^ ^]^ Follow‐up studies are needed to investigate the specific molecular mechanisms by which ARG1 inhibitors suppress CRC growth in vivo. Our study found a significant reduction of lysine Kbhb in CRC tissues compared to matched adjacent normal tissues, with only slight increases in Kac, Kla, and O‐GlcNAc detected. Further exploration of the antagonistic or synergistic interactions between Kbhb and these modifications in CRC may provide insights into the role of PTMs in CRC progression. Finally, it remains to be determined whether ARG1 Kbhb drives reprogramming of arginine metabolism is a common phenomenon in other cancers and whether other cancers could be targeted ARG1 Kbhb with BHB.

**Figure 11:**
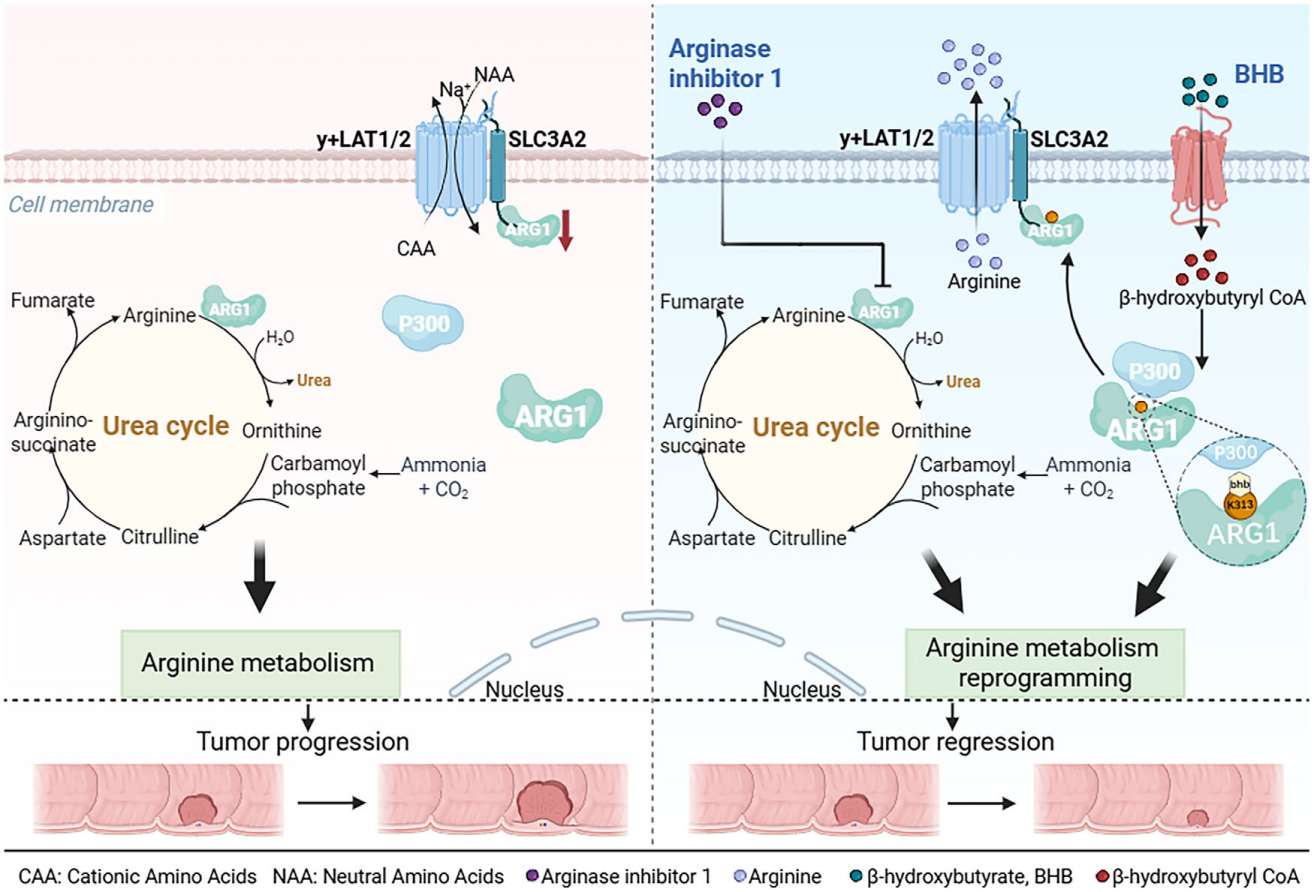
Schematic summarization of key findings presented in this study. Created in BioRender. Lin, C. (2025) https://BioRender.com/3iushyq.

Our research indicated that CRC was associated with activated arginine metabolism, accompanied by elevated arginine levels and the high expression of ARG1. Furthermore, Kbhb modification of ARG1 by BHB triggered arginine metabolic reprogramming by enhancing the interaction of ARG1 with SLC3A2 in CRC cells, which promoted the efflux of arginine, thereby decreasing intracellular arginine levels and suppressing tumorigenicity in a catalytic‐activity‐independent manner. Moreover, administration of BHB with ARG1‐i1 in combination based on the conventional enzymatic role and nonenzymatic‐metabolic function of ARG1 specifically suppressed the growth of the tumor. This study provided a novel perspective on the mechanism of CRC by exploring arginine metabolic reprogramming and Kbhb modification, and identified potential therapeutic interventions for the treatment of CRC (**Figure** **11**).

In conclusion, our work focusing on the ARG1‐Kbhb/P300/SLC3A2 signaling axis may offer valuable insights into arginine metabolism reprogramming pertaining to the pathogenesis and identification of PTMs targets in CRC. Meanwhile, we uncovered an effective combination therapy that encompassed the conventional enzymatic role and nonenzymatic‐metabolic function of ARG1, bridging the connection between arginine metabolic reprogramming and PTMs.

## 4 Experimental Section

### Ethics Statement

Fresh primary CRC specimens with paired noncancerous colorectal tissues were obtained from the Tumor Tissue Bank of Nanfang Hospital, with the approval of the Human Ethics Committee of the Nanfang Hospital, Southern Medical University (Guangzhou, China) (project numbers NFEC‐2024‐334). All patients signed an informed consent form approved by the local institutional review board.

### Bioinformatics Analysis

This study utilized the publicly available dataset GSE132465 in the Gene Expression Omnibus (GEO) (https://www.ncbi.nlm.nih.gov/gds), which encompassed single‐cell RNA sequencing (scRNA‐seq) data of tumor samples from 23 Korean patients with primary CRC and 10 matched normal mucosa samples. To guarantee data quality, high‐quality cells were initially screened based on criteria such as unique molecular identifiers (UMI), the number of genes (nGene), and the percentage of mitochondrial genes, and 67,296 cells were obtained after the initial screening. Further, the cells were initially clustered and annotated through Seurat's multi‐canonical correlation analysis (multiCCA) and robust clustering analysis (RCA) pipelines, and cells that were inconsistent between the two methods were eliminated. After defining the global cell types, cells with a number of genes exceeding the outliers were removed to eliminate potential doublets, and ultimately, 63,689 cells were retained for subsequent analysis. After quality control, each cell cluster was annotated with known marker genes to identify the cell types. Once the marker gene annotation was completed, epithelial cells were extracted, and the gene expression differences between the primary CRC and normal mucosa samples were compared using the FindMarkers function of the Seurat package to screen for DEGs for further analysis. Functional analyses included GSEA and GO enrichment analysis. GSEA was employed to assess the cumulative expression trend of specific gene sets in tumor and normal phenotypes to disclose tumor‐related biological processes. GO enrichment analysis annotated the functions of differential genes from three aspects: molecular function, cellular component, and biological process. The data above were analyzed with R (version 4.3.2).

### Tumor Tissue Samples

Fresh primary CRC specimens with paired noncancerous colorectal tissues were obtained from the Tumor Tissue Bank of Nanfang Hospital. In total, 53 patients diagnosed with CRC and undergoing surgical resection at Nanfang Hospital of Southern Medical University were included in this study. Detailed information pertaining to these CRC patients is available in Table S1 (Supporting Information).

### Histological and Immunohistochemistry Analysis

Tissues were treated with 4% paraformaldehyde and embedded in paraffin. Then, the slides were prepared using 2.5 µm‐thick continuous sections. The slides were roasted at 70 °C for 1 h and then dewaxed with xylene three times for 10 min. The slides were soaked in 100%, 100%, 95%, 90%, 80%, and 70% anhydrous ethanol for 1 min successively and then washed with ddH_2_O for 1 min. Subsequently, hematoxylin‐eosin (HE) staining was performed.

For immunohistochemistry analysis with paired CRC and adjacent normal tissues, antigen repair was performed with sodium citrate solution (pH 6.0) (BOSTER, AR0024, Wuhan, China) for 3 min at 110 kPa. The sections were incubated overnight with 1:400 dilutions of the indicated primary antibodies overnight at 4 °C. This was followed by incubation with secondary antibody conjugated with 3,3′‐diaminobenzidine (DAB) chromogen. Samples were scanned by a digital pathology scanner (3DHISTECH, DX300, Shandong, China). Detailed information about the antibodies used in this study is available in Table S2 (Supporting Information). Multiplication of staining intensity and the percentage of tumor‐positive cells was calculated as immunochemical staining indexes. The IHC staining was scored semi‐quantitatively as 0 (no staining), 1 (weak staining, light yellow), 2 (moderate staining, yellowish brown), and 3 (strong staining, brown) based on the intensity of staining.

### Cell Culture and Treatment

The normal human colon epithelial cell line NCM460, the CRC cell lines (HCT116, RKO, SW480, SW620, HCT‐8, LS174T, HCT‐15, DLD‐1 and HT‐29) and human embryonic kidney 293T cells (HEK 293T) were obtained from the Cell Bank of the Chinese Academy of Sciences (Shanghai, China). NCM460 and CRC cell line were cultured in RPMI‐1640 (Pricella, PM150110, Wuhan, China) supplemented with 10% fetal bovine serum (Vazyme, F101‐01, Nanjing, China), while HEK 293T were cultured in DMEM (Pricella, PM150210, Wuhan, China) with 10% fetal bovine serum in a 5% CO2 incubator at 37 °C. According to viability assays, the final concentrations of Arginase inhibitor 1 (ARG1‐i1, dissolved in DMSO at a working concentration of 10 µmol L^−1^) (MedChemExpress, HY‐15775, Shanghai, China), β‐hydroxybutyrate (BHB, dissolved in serum‐free medium at a working concentration of 30 mmol L^−1^ and filtered through sterile syringe filters before use) (BHB, Macklin, S832832, USA) (0.2 µm Whatman Puradisc sterile PES syringe filters, Cytiva) and A485 (dissolved in DMSO at a working concentration of 10 nmol L^−1^) (MedChemExpress, HY‐107455, USA) were used in this study.

### Cell Transfection

The siRNA sequences of ARG1 and P300 were designed by GeneCreate (Wuhan, China) and provided in Table S3 (Supporting Information). The coding sequences for full‐length human ARG1 and mutant ARG1 (ARG1^K39R^ and ARG1^K313R^) were subcloned into the pcDNA3.1‐Flag vector and validated by OBiO Technology (Shanghai, China). The coding sequences for ARG1 and P300 were subcloned into the pcDNA3.1‐Flag vector and pCMV‐HA vector, respectively, and validated by Kidan Biosciences (Guangzhou, China). The coding sequences for truncated ARG1 (ARG1^WT^, ARG1^181‐322aa^, ARG1^1‐240aa^, ARG1^1‐180aa^) and truncated SLC3A2 (SLC3A2^WT^, SLC3A2^185‐630aa^, SLC3A2^△185‐205aa^, SLC3A2^1‐184aa^) were subcloned into pCDH‐Flag vector and pCDH‐HA vector, respectively, and validated by Kidan Biosciences. Lipofectamine 3000 (Invitrogen, L3000001, USA) was employed to transfect the siRNA or plasmid following the supplier's manufacturer's instructions.

### RNA Extraction and RT‐qPCR

Total RNA extraction, cDNA reverse transcription, and RT‐qPCR analysis were carried out according to established protocols as previously described.^[^ ^78^ ^]^ Sequences of the specific forward and reverse primers used in this study are listed in Table S4 (Supporting Information).

### Western Blot

Protein was lysed using RIPA lysis buffer (KeyGEN Biotechnology Co., KGB5203‐100, Nanjing, China) with protease inhibitor cocktails and phosphatase inhibitor cocktail (Epizyme Biotech, GRF101, GRF102, Shanghai, China) and then centrifuged. The BCA Protein Assay Kit (KeyGEN Biotechnology Co., KGB2101‐500, Nanjing, China) was used for protein determination. Immunoblotting was performed with primary antibodies and secondary horseradish peroxidase‐conjugated antibodies. Blots were visualized with the Tanon‐5200 Chemiluminescent Imaging System (Tanon Science & Technology). Primary antibodies (1:1000) used were listed in Table S2 (Supporting Information).

### 5‐Ethynyl‐2′‐deoxyuridine (EdU) Assay

EdU assay was conducted with BeyoClick EdU Cell Proliferation Kit with Alexa Fluor 594 (Beyotime, C0078S, Shanghai, China). Briefly, the treated cells were seeded in 6‐well plates with cover glasses (NEST Biotechnology Co. Ltd., 801009, Wuxi, China). After washing in PBS, EdU solution was used to incubate cells for 2 h. Cell nuclei were then stained with Hoechst 33342 solution. After EdU staining, cells were observed with a fluorescence microscope (ZEISS, LSM980, Oberkochen, Germany). The percentage of Edu‐positive cells was calculated as the number of Edu‐positive cells out of the total number of cells.

### Cell Proliferation, Migration, and Invasion Assay

Cell Counting Kit‐8 (CCK‐8) (APExBIO, K1018, USA) and colony formation assay were used to assess cell proliferation ability. For the CCK‐8 assay, the treated cells were seeded in 96‐well plates and incubated with 10 µL of CCK‐8 solution at 37 °C for 1.5 h. The optical density (OD) value of each well at 450 nm wavelength was measured using a microplate reader (BioTek). For the colony formation assay, the treated HCT116 and RKO cells were seeded in 6‐well plates (1×10^3^ cells per well) and cultured for 1 week. After being washed three times with cold PBS, colonies were stained with hematoxylin solution overnight at room temperature and then photographed.

The cell migration assay was performed in a 24‐well transwell chamber (8 µm pore size, SAINING LIFE SCIENCES, 1102130, China). HCT116 and RKO cells (2×10^5^ /300 µL) with different treatments were cultured in the upper compartment in serum‐free medium, while the lower compartment was mixed with 20% complete medium. After incubation at 37 °C for 24 h (HCT116) or 48 h (RKO), the cells were fixed with 4% paraformaldehyde, stained with crystal violet, and then photographed with a microscope. The invasion assay was implemented similarly, with the coating of the filters with Matrigel (Corning, 356234, USA).

### Liquid Chromatograph Mass Spectrometer (LC‐MS) Analysis

HCT116 cells were treated with or without BHB for 48h, then the cells were harvested with IP lysis buffer containing 1% leagene PMSF, 1% protease, and Phosphatase Inhibitor Cocktail on ice for 30 min. An anti‐Kbhb antibody was used to pull down ARG1, and an anti‐ARG1 antibody was used to pull down SLC3A2, respectively. LC‐MS analyses were carried out as follows: SDS‐PAGE gels were stained with Coomassie blue and then cut into gel blocks, followed by trypsin digestion. Next, the resulting tryptic peptides were purified using Zeba Spin Desalting Columns and then analyzed on a Q‐Exactive mass spectrometer (Thermo Scientific). Next, MS/MS spectra were collected for the selected precursor ions within a mass isolation window of 0.02 Da. Subsequently, the spectral data were searched via Proteome Discoverer 2.1 against the UniProt protein database. Following the database search, peptide spectrum matches (PSMs) for ARG1 or SLC3A2 were obtained.

### Co‐Immunoprecipitation (Co‐IP)

Samples were lysed in IP lysis buffer (KeyGEN Biotechnology Co., KGB5202‐100, Nanjing, China) containing 1% leagene PMSF, 1% protease, and Phosphatase Inhibitor Cocktail on ice for 30 min. Subsequently, the samples were centrifuged, and the supernatant was collected, followed by incubation with primary antibodies and Protein A/G Magnetic Beads (Selleck, B23202, USA) with gentle rocking overnight at 4 °C. The next day, the mixture was pelleted, washed three times with PBST buffer and three times with PBS buffer, and then analyzed by Western blot.

### GST Pull‐Down Assay

The coding sequences for truncated SLC3A2 (SLC3A2^WT^, SLC3A2^1‐184aa^, SLC3A2^206‐630aa^) were subcloned into the pGEX‐4T‐1‐GST vector and validated by Kidan Biosciences. The recombinant plasmids pGEX‐4T‐1‐GST, GST‐SLC3A2^WT^, GST‐SLC3A2^1‐184aa,^ and GST‐SLC3A2^206‐630aa^ were transformed into E. coli (DL‐BL21/DE3), respectively, to induce expression of recombinant proteins in the presence of 0.5 mM IPTG at 16 °C overnight. Purification of GST‐tagged (GST, GST‐SLC3A2^WT^, GST‐SLC3A2^1‐184aa,^ and GST‐SLC3A2^206‐630aa^) recombinant proteins was performed utilizing the Beaver Beads GSH and Beaver Beads kit, respectively. For GST pull‐down assay, 20 µg of purified GST, GST‐SLC3A2^WT^, GST‐SLC3A2^1‐184aa^ and GST‐SLC3A2^206‐630aa^ and cell lysate of HCT116 cells transfected with ARG1 plasmid were incubated with 100 µL GST beads for 2 h at 4 °C. After extensive washing, samples were suspended in the reducing SDS loading buffer, boiled for 10 min, and subjected to SDS‐PAGE followed by immunoblotting. The primary antibodies used include anti‐GST and anti‐ARG1.

### Arginine Content and Arginase Activity Assay

Arginine content was assessed by the Arginine content assay kit (Solarbio, BC5630, Beijing, China) following the supplier's procedure. In brief, 5×10^6^ CRC cells or 0.1 g of tissues were lysed in 1 mL ice‐cold arginine extraction solution. After centrifugation at 12000 g for 10 min, the supernatant was collected and added to a 96‐well plate with the reaction mix. Absorbance at 525 nm was measured after reaction for 20 min on ice.

Arginase activity was assessed employing Arginase Activity Colorimetric Assay Kit (Abcam, ab180877, UK) following the manufacturer's instructions. Briefly, 1×10^6^ CRC cells were lysed in 100 µl ice‐cold assay buffer. After centrifugation at 10000 g for 5 min, the supernatant was collected and added to a 96‐well plate with reaction mix (arginase assay buffer, arginase substrate) for 20 min at 37 °C. Then add 50 µL of arginase reaction mix (arginase assay buffer, arginase enzyme mix, arginase developer, arginase converter enzyme, and OxRred probe) into each sample. Absorbance at 570 nm was measured in a kinetic mode for 10–30 min at 37 °C.

### Immunofluorescence (IF)

The treated HCT116 cells and RKO cells were seeded in the glass bottom cell culture dishes (NEST Biotechnology Co. Ltd., 801002, Wuxi, China) and then fixed in 4% paraformaldehyde for 15 min. Subsequently, the cells were washed with PBS before a 1 h incubation with 5% normal goat serum at room temperature to block nonspecific binding of antibodies, following an overnight cultivation at 4 °C with the primary antibodies ARG1 and SLC3A2 in block buffer. Next, the dishes were washed with PBS, and the secondary antibodies were added to the dishes and incubated for 1 h at room temperature. Subsequently, nuclei were stained with 4', 6‐diamidino‐2‐phenylindole (DAPI) (Beyotime, C1005, Shanghai, China). Images were captured with a fluorescence microscope (ZEISS, LSM980, Oberkochen, Germany).

### Molecular Docking Analysis

Rigid protein–protein docking was performed between ARG1 and SLC3A2 to investigate the relationships by using GRAMM‐X (http://gramm.compbio.ku.edu/). The protein structural domains of ARG1 and SLC3A2 were obtained from the Protein Data Bank PDB database(http://www.rcsb.org/). Pymol (Version 3.0.3) and PDBePISA (https://www.ebi.ac.uk/pdbe/pisa/) were used to investigate protein‐protein interactions and further visual analysis.

### Animal Model

All animal experiments in the study were approved by the Animal Care and Use Committee of Southern Medical University. Five‐week‐old BALB/c nude mice were purchased from Zhiyuan Biology (animal certificate numbers: SCXK 2021‐0057, Guangzhou, China). All animals were maintained under a 12 h light/dark schedule with free access to purified water and food throughout the experiment. For the xenograft experiment, HCT116 cells marked with luciferase (8×10^6^ /150 µL) were injected subcutaneously into the mice. Body weight and tumor volumes were determined every 4 days and tumor volumes were calculated according to the following formula: Volume = (length×width^2^)/2. Tumors were monitored by injecting D‐Luciferin potassium (150 mg kg^−1^, 5 min before imaging, AmBeed, A162108, USA) intraperitoneally into the mice, followed by bioluminescence analysis using an In Vivo Imaging System (Bruker, BUFB20041, USA). All mice were humanely sacrificed after 25 days, and their tumors and tissues were dissected, weighed, and stored for subsequent experiments. For the drug administration experiment, mice received equal volume (100 µL) of saline, β‐hydroxybutyrate (2 g kg^−1^ day^−1^), or ARG1‐i1 (10 mg kg^−1^ day^−1^) once daily, by gavage administration.

### Statistical Analysis

Statistical analyses were conducted in SPSS 29.0 (IBM, Armonk, USA), and the results were visualized using GraphPad Prism version 10 (GraphPad, La Jolla, USA). All data in the text and graphs were expressed as the mean ± standard deviation. Student's t‐test was performed for continuous variables between the two groups. Statistical differences among multiple groups were examined using one‐way ANOVA with post hoc Fisher's LSD test. A p‐value less than 0.05 was considered statistically significant (^*^ *p* < 0.05, ^**^ *p* < 0.01, ^***^ *p* < 0.001).

## Conflict of Interest

The authors declare no conflict of interest.

## Author Contributions

C.L., Z.L., X.Z., and W.Z. contributed equally to this work. C.L. and J.L. designed the study. C.L., Z.L., X.Z., and W.Z. carried out most experiments. C.L., Z.L., and X.L. performed bioinformatic analysis. C.L. and J.Z. conducted the statistical analysis. C.L. wrote the manuscript. J.L. revised the paper. All authors read and approved the final manuscript.

## Supporting information



Supporting Information

## Data Availability

The data that support the findings of this study are available from the corresponding author upon reasonable request.
