# Supplementary material for: Abnormal β‐Hydroxybutyrylation Modification of ARG1 Drives Reprogramming of Arginine Metabolism to Promote the Progression of Colorectal Cancer
Source: Adv Sci (Weinh). 2025 Jul 11;12(38):e02402. doi: 10.1002/advs.202502402 (PMC12520464; doi:10.1002/advs.202502402)
Supplement: Supplementary file 1 — Supporting Information [file ADVS-12-e02402-s001.docx]

Figure S1


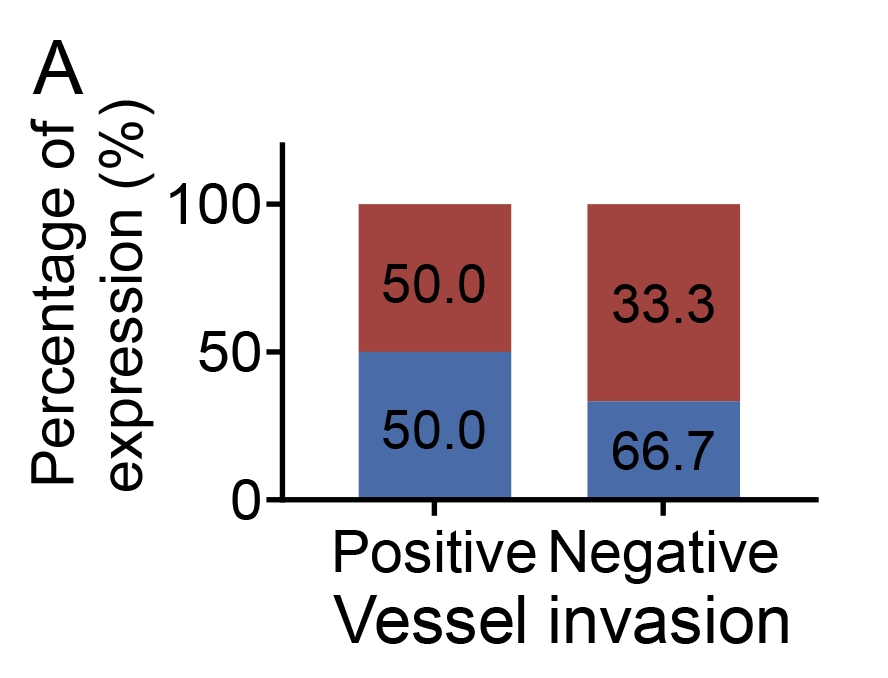


**Figure S1. The Vessel invasion based on ARG1 levels.**

Figure S2


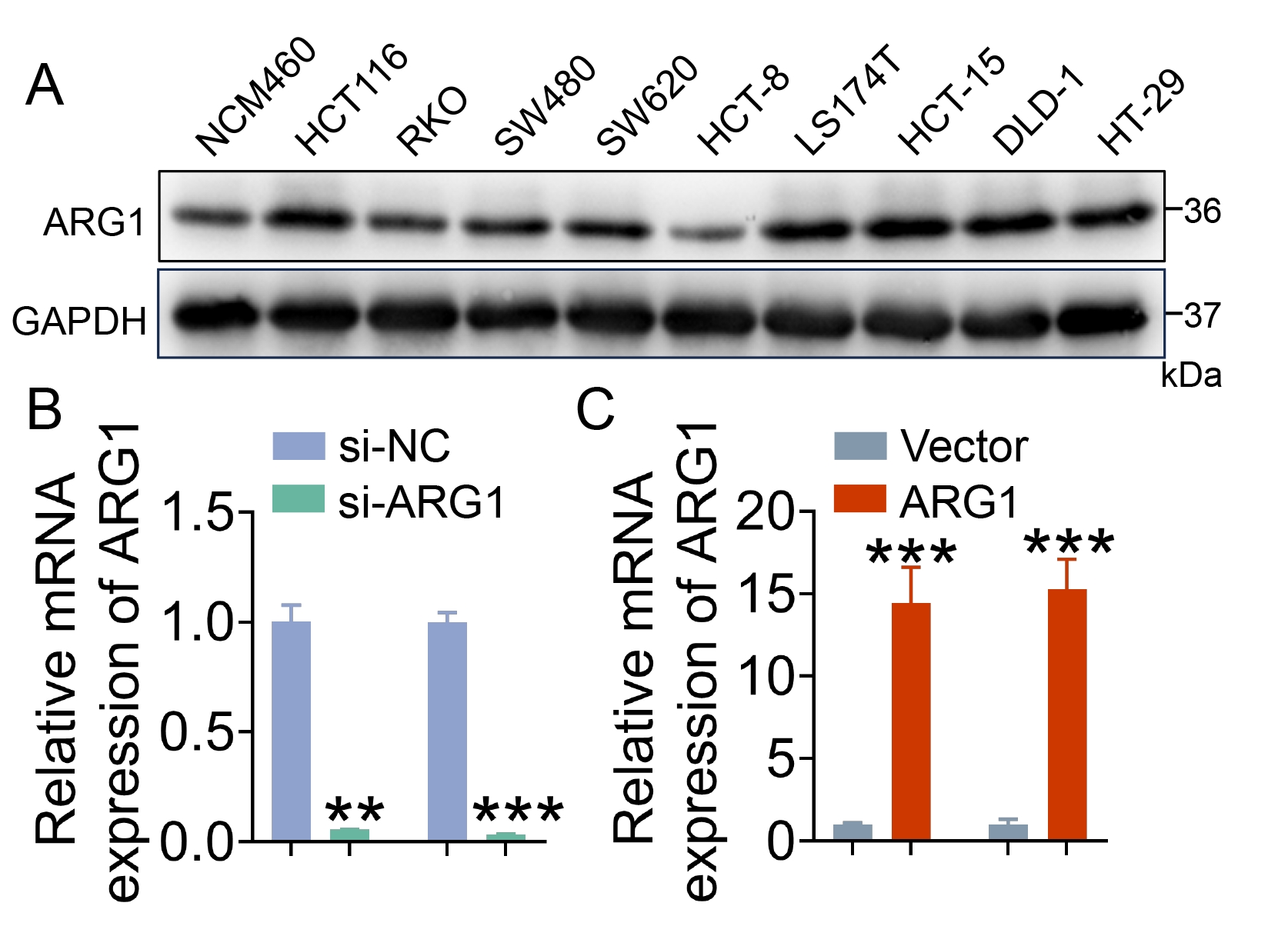


**Figure S2. Protein and mRNA expression of ARG1 in the indicated cells.**

**A)** Protein levels of ARG1 in the normal human colon epithelial cell lines (NCM460) and CRC cell lines (HCT116, RKO, SW480, SW620, HCT-8, LS174T, HCT-15, DLD-1 and HT-29). **B)** mRNA levels of ARG1 in HCT116 and RKO cells transfected with si-NC or si-ARG1. **C)** mRNA levels of ARG1 in HCT116 and RKO cells transfected with vector or ARG1 plasmid. Data are presented as means ± SD; n=3. **p < 0.01, ***p < 0.001, versus the si-NC or Vector group.

Figure S3


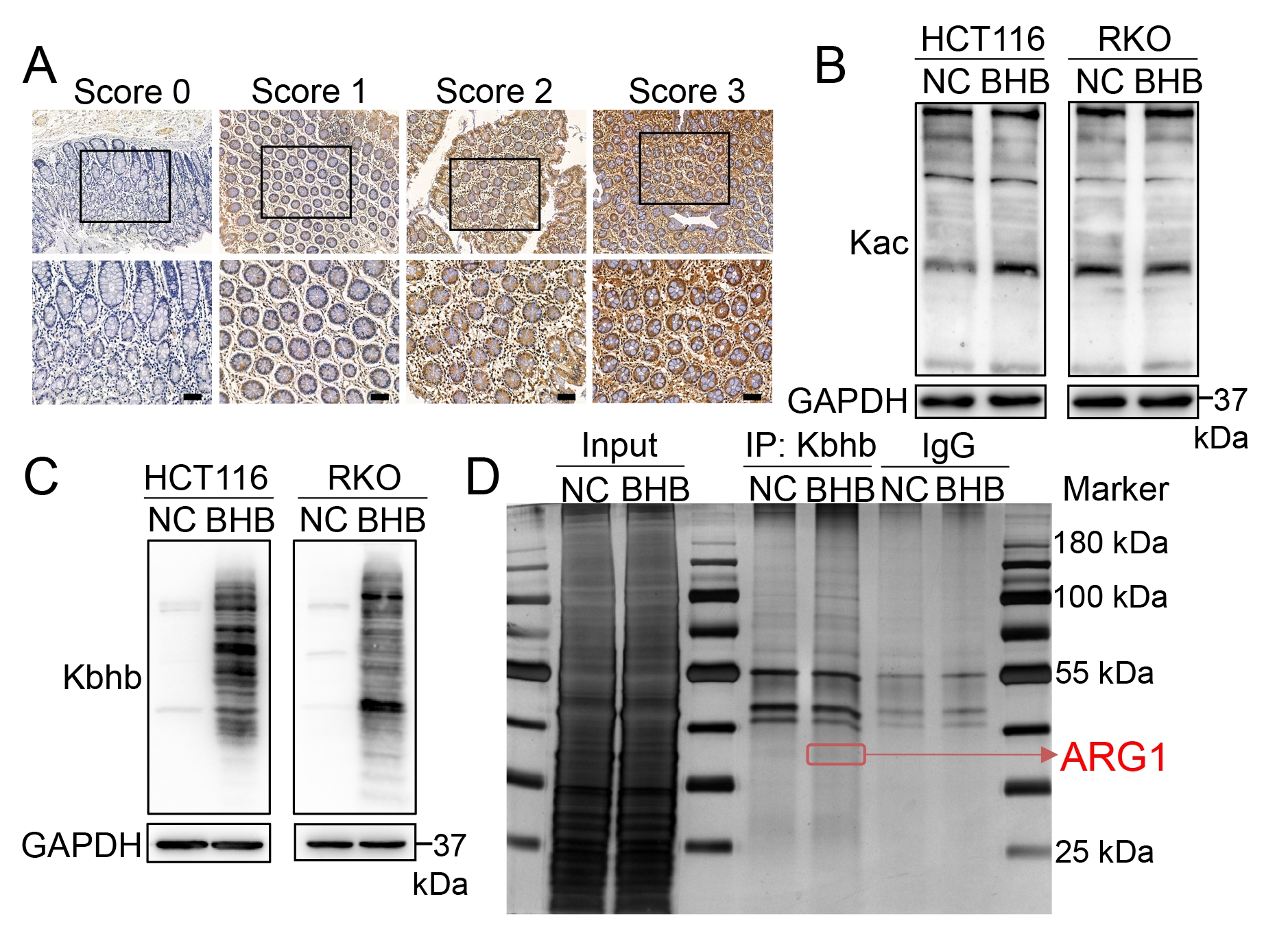


**Figure S3. BHB promoted Kbhb of ARG1.**

**A)** Scores indicated Kbhb protein levels in representative tumor tissues containing 52 patient specimens. Scale bar: 50 μm. **B-C)** Western blotting analysis showing the levels of Kac **(B)** and Kbhb **(C)** in HCT116 and RKO cells treated with or without BHB. n=3. **D)** Analysis of proteins in the Input and immunoprecipitated with anti-Kbhb groups was conducted using SDS/PAGE followed by coomassie blue staining.

Figure S4


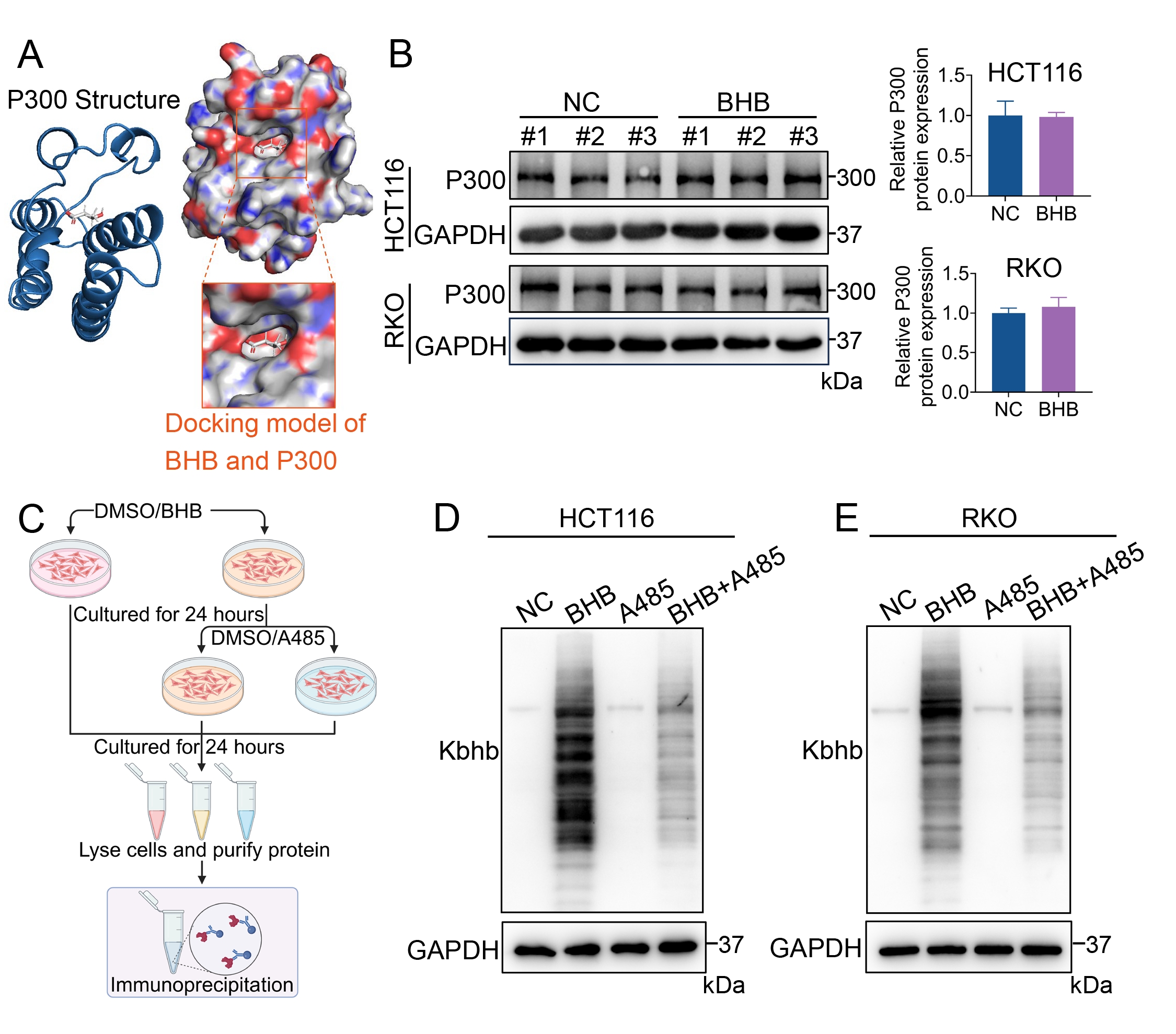


**Figure S4. P300 Catalyzes the Kbhb of ARG1.**

**A)** Docking models were performed based on the P300 crystal structure and BHB compounds. **B)** Western blotting analysis showing the levels of P300 in HCT116 and RKO cells treated with or without BHB. n=3. **C)** Schematic of HCT116 and RKO cells supplemented with or without BHB and A485, followed by immunoprecipitation. Created in BioRender. Lin, C. (2025) <https://BioRender.com/cyx3a07.> **D-E)** Western blotting analysis showing the levels of Kbhb in HCT116 **(D)** and RKO **(E)** cells treated with or without BHB and A485. n=3.

Figure S5


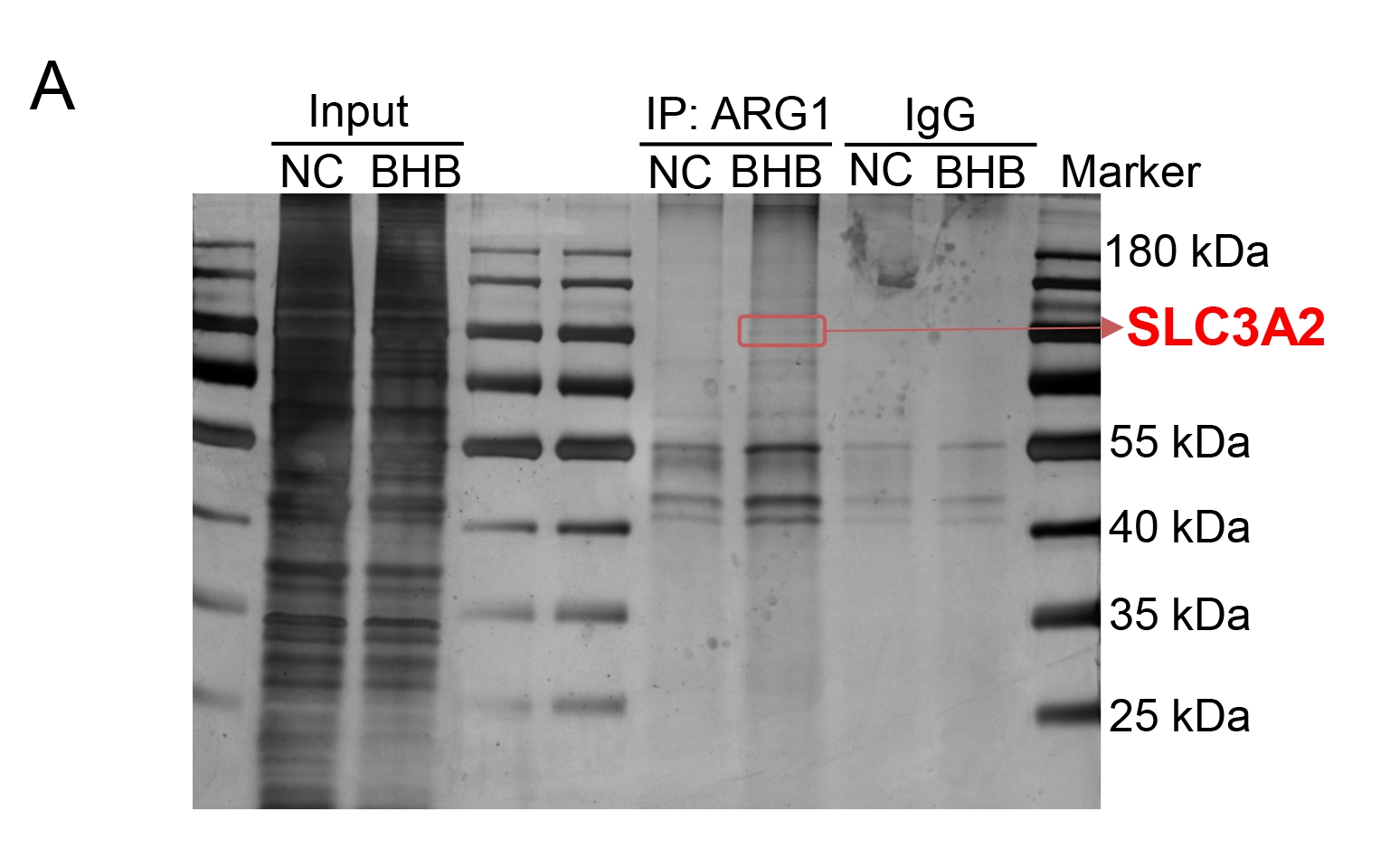


**Figure S5. Analysis of proteins in the Input and immunoprecipitated with anti-ARG1 groups was conducted using SDS/PAGE followed by coomassie blue staining.**

Figure S6


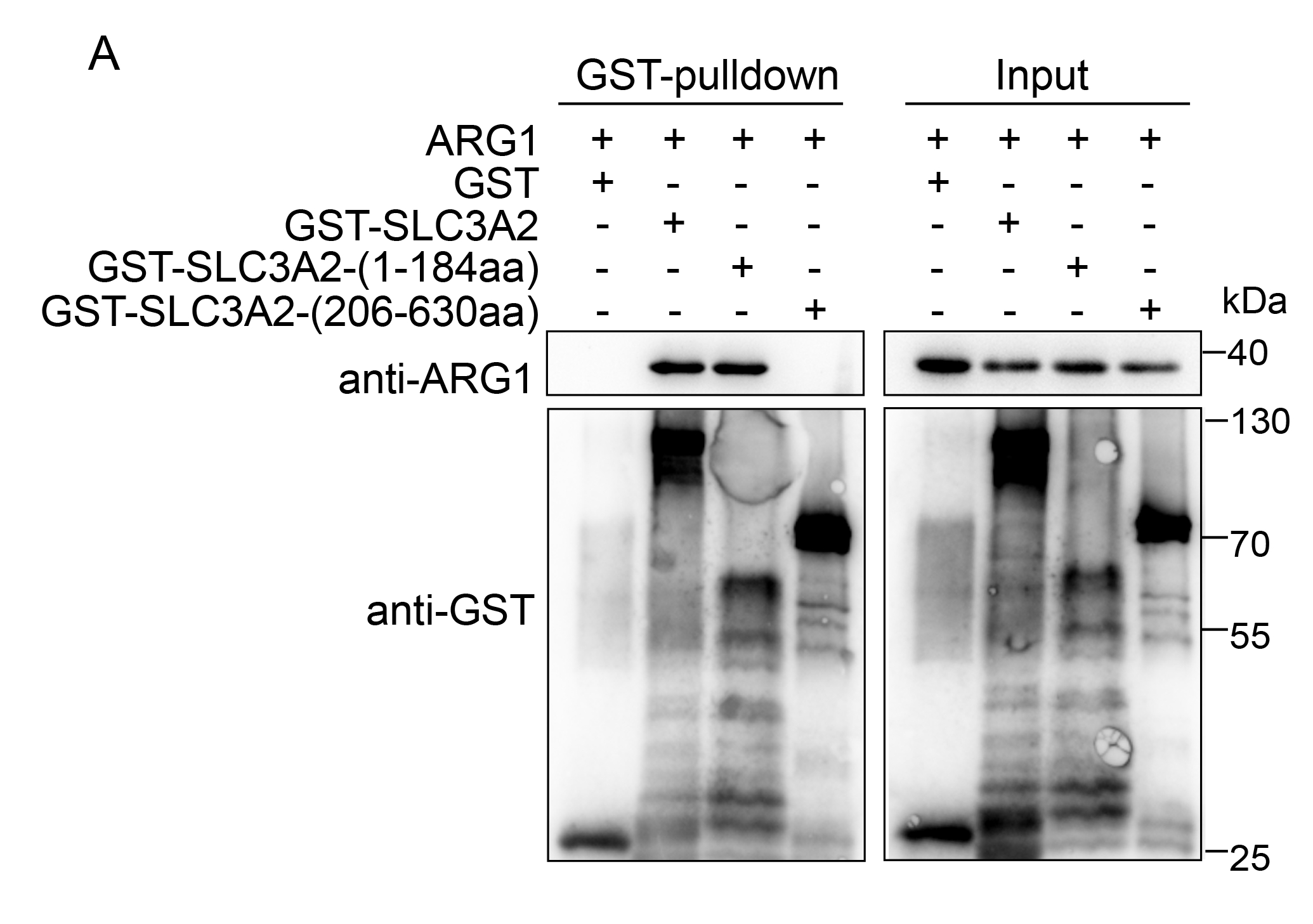


**Figure S6.** **Direct binding of ARG1 to the intracellular domain of SLC3A2 using GST pull down assay.**

Figure S7


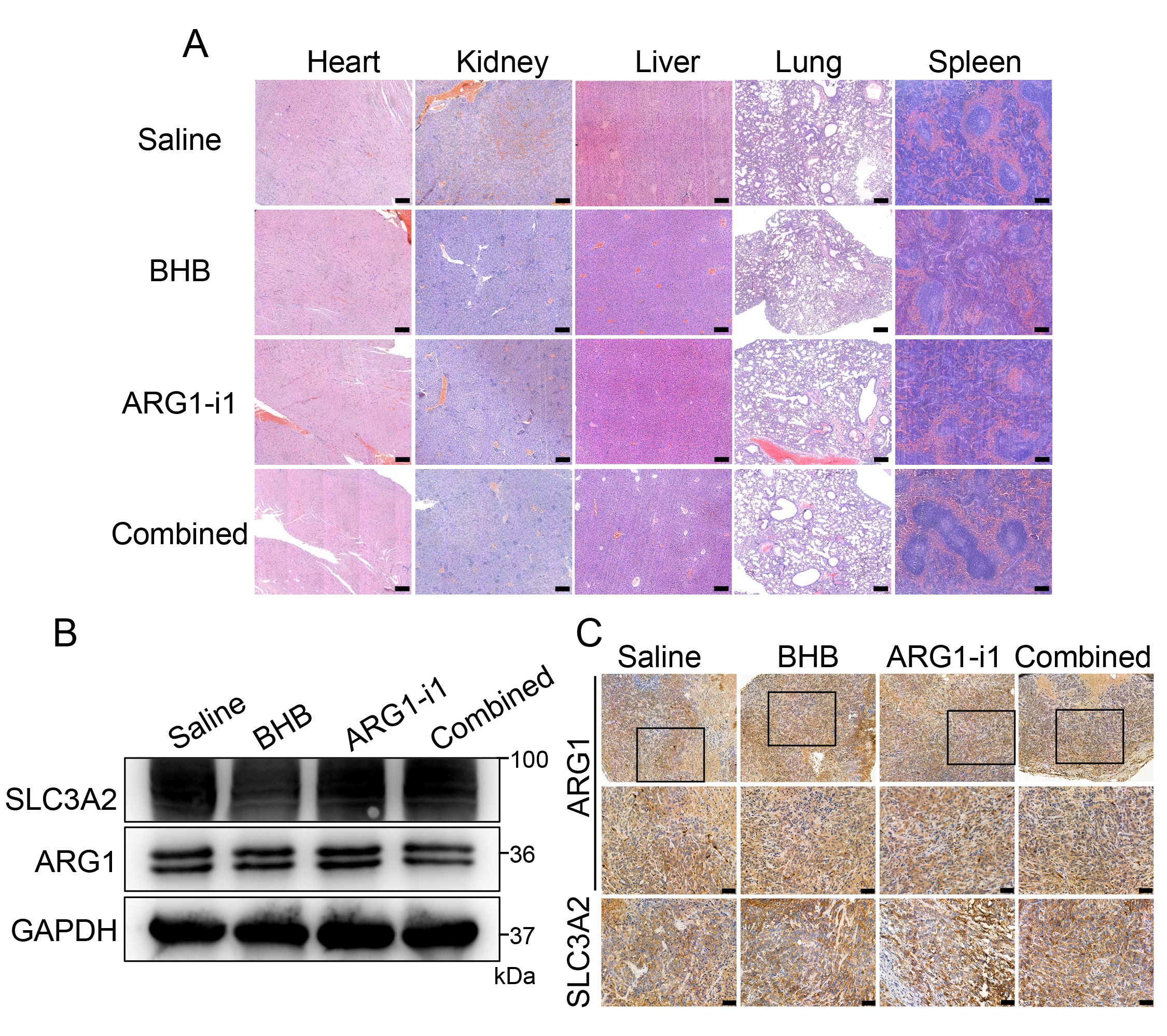


**Figure S7. Bio-safety of small molecule compounds (BHB and ARG1-i1) and expression of SLC3A2 and ARG1 in BALB/c nude mice.**

**A)** HE staining of heart, kidney, liver, lung and spleen in mouse model treated with saline or BHB or ARG1-i1 or BHB and ARG1-i1. Scale bar: 200 µm. **B)** Western blotting analysis showing the levels of SLC3A2 and ARG1 in BALB/c nude mice for indicated groups. **C)** Representative images of SLC3A2 and ARG1 IHC staining in different treatment groups. Scale bar: 50 µm. n=5.

Table S1. Clinicopathologic characteristics of patients enrolled for sample examination in 53 CRC.

| **No.** | **Age(y)** | **Gender** | **Tumor size** | **Tumor site** | **Grade** | **T stage** | **Blood vessel invasion** |
| --- | --- | --- | --- | --- | --- | --- | --- |
|  |  |  | **(cm)** |  |  |  |  |
| 1 | 60 | M | 3.5 | Colorectum | High | T3 | Positive |
| 2 | 40 | F | 4.5 | Colorectum | Low | T3 | Negative |
| 3 | 39 | M | 4.5 | Colorectum | Low | T3 | Negative |
| 4 | 50 | F | 5.1 | Colon | High | T3 | Negative |
| 5 | 41 | M | 3 | Colon | High | T3 | Negative |
| 6 | 60 | M | 4.5 | Rectum | Low | T3 | Negative |
| 7 | 55 | M | 4 | Colon | High | T3 | Positive |
| 8 | 52 | F | 5 | Colon | Low | T3 | Negative |
| 9 | 75 | M | 4.5 | Colon | Low | T3 | Negative |
| 10 | 64 | M | 5 | Rectum | High | T3 | Negative |
| 11 | 56 | M | 2.5 | Colorectum | Low | T2 | Negative |
| 12 | 39 | F | 4.6 | Colon | High | T3 | Positive |
| 13 | 68 | F | 3 | Colon | High | T3 | Negative |
| 14 | 35 | M | 7.4 | Colon | Low | T4 | Negative |
| 15 | 68 | M | 11.5 | Colon | High | T4 | Negative |
| 16 | 85 | M | 3 | Colon | High | T3 | Negative |
| 17 | 64 | F | 4 | Colon | High | T3 | Negative |
| 18 | 59 | F | 5 | Colon | Low | T2 | Positive |
| 19 | 67 | M | 5 | Colon | High | T4 | Negative |
| 20 | 72 | M | 3 | Rectum | High | T4 | Negative |
| 21 | 47 | M | 6.5 | Colorectum | High | T4 | Negative |
| 22 | 49 | M | 6.5 | Rectum | High | T3 | Negative |
| 23 | 57 | F | 5 | Colon | High | T3 | Negative |
| 24 | 73 | F | 4 | Rectum | Low | T2 | Negative |
| 25 | 74 | F | 2.5 | Rectum | Low | T3 | Positive |
| 26 | 66 | M | 4.5 | Colorectum | Low | T3 | Positive |
| 27 | 80 | F | 8 | Colon | Low | T4 | Positive |
| 28 | 58 | F | 4.2 | Colon | High | T3 | Negative |
| 29 | 56 | F | 4 | Rectum | Low | T3 | Negative |
| 30 | 57 | F | 4 | Colorectum | High | T3 | Negative |
| 31 | 55 | M | 4 | Rectum | High | T1 | Negative |
| 32 | 51 | M | 5.5 | Colon | Low | T3 | Positive |
| 33 | 42 | M | 3.2 | Colon | High | T3 | Negative |
| 34 | 85 | F | 6 | Colon | High | T3 | Negative |
| 35 | 48 | M | 6.2 | Colon | High | T3 | Negative |
| 36 | 60 | M | 6 | Colon | Low | T3 | Negative |
| 37 | 70 | M | 3.5 | Colon | Low | T4 | Negative |
| 38 | 35 | F | 5 | Colon | High | T2 | Negative |
| 39 | 61 | M | 11 | Colon | High | T3 | Negative |
| 40 | 70 | M | 4 | Colon | Low | T3 | Negative |
| 41 | 64 | M | 5 | Colon | High | T3 | Negative |
| 42 | 74 | M | 4.5 | Rectum | High | T4 | Positive |
| 43 | 71 | M | 3.5 | Rectum | High | T2 | Negative |
| 44 | 36 | F | 2.5 | Rectum | High | T4 | Negative |
| 45 | 59 | M | 5.5 | Rectum | High | T3 | Negative |
| 46 | 73 | M | 3 | Colorectum | High | T3 | Positive |
| 47 | 66 | M | 4 | Rectum | High | T4 | Positive |
| 48 | 71 | F | 10 | Colon | High | T4 | Negative |
| 49 | 84 | M | 5 | Colon | High | T3 | Negative |
| 50 | 68 | M | 4 | Rectum | Low | T3 | Positive |
| 51 | 70 | M | 4 | Rectum | Low | T2 | Negative |
| 52 | 51 | M | 2.5 | Colon | Low | T3 | Positive |
| 53 | 79 | M | 4 | Rectum | High | T3 | Positive |

Table S2. The antibodies used in this study.

| **Antibody** | **Supplier** | **Catalogue** | **Host** |
| --- | --- | --- | --- |
| Anti-ARG1 | Proteintech | 16001-1-AP | Rabbit |
| Anti-ARG1 | Proteintech | 66129-1-Ig | Mouse |
| Anti-GAPDH | Proteintech | 60004-1-Ig | Mouse |
| Anti-Acetyllysine | PTMBIO | PTM-101 | Mouse |
| Anti-β-Hydroxybutyryllysine | PTMBIO | PTM-1201RM | Rabbit |
| Anti-D-Lactyl Lysine | PTMBIO | PTM-1429RM | Rabbit |
| Anti-O-Linked N-Acetylglucosamine | PTMBIO | PTM-955RM | Rabbit |
| Anti-SLC3A2 | Abcam | Ab307587 | Rabbit |
| Anti-SLC3A2 | CST | 47213S | Rabbit |
| Anti-P300 | CST | D8Z4E | Rabbit |
| Anti-P300 | CST | E8S2V | Rabbit |
| Anti-Flag | Proteintech | 20543-1-AP | Rabbit |
| Anti-HA | Zenbio | 390001 | Rabbit |
| Anti-GST | ABMART | M20007F | Mouse |
| HRP Conjugated AffiniPure Goat Anti-mouse IgG (H+L) | Boster | BA1050 | Mouse |
| HRP Conjugated AffiniPure Goat Anti-rabbit IgG (H+L) | Boster | BA1054 | Rabbit |
| Dylight 549, Goat Anti-Rabbit IgG(H+L) | EarthOx | E032320-01 | Rabbit |
| Dylight 649, Goat Anti-Mouse IgG(H+L) | EarthOx | E032610-01 | Mouse |
| DAPI Staining Solution | Beyotime | C1005 |  |

Table S3. Targeted sequences of siRNA and of ARG1 used in our study.

| **siRNA** | **Sequence** |
| --- | --- |
| siRNA-ARG1 | 5′- GAACAAGAGUGUGAUGUGATT -3′ |
| siRNA-P300 | 5′- UUUAUGUAAACGCGACCUCTT -3′ |

Table S4. Primers for RT-qPCR.

| **Gene** |  | **Sequence** |
| --- | --- | --- |
| ARG1 | Forward | 5′- GTGGAAACTTGCATGGACAAC -3′′ |
|  | Reverse | 5′- AATCCTGGCACATCGGGAATC -3′ |
| GAPDH | Forward | 5′- TGCACCACCAACTGCTTAGC -3′ |
|  | Reverse | 5′- GGCATGGACTGTGGTCATGAG -3′ |
